# **X**-CoOTe (**X** = S, Se, and P) with Oxygen/Tellurium Dual Vacancies and Banana Stem Fiber-Derived Carbon Fiber **a**s Battery-Type Cathode and Anode Materials for Asymmetric Supercapacitor

**DOI:** 10.1021/acsami.3c18205

**Published:** 2024-04-02

**Authors:** Mani Sakthivel, Kuo-Chuan Ho

**Affiliations:** † Department of Chemical Engineering, National Taiwan University, Taipei 10617, Taiwan; ‡ Advanced Research Center for Green Materials Science and Technology, National Taiwan University, Taipei 10617, Taiwan; § Institute of Polymer Science and Engineering, National Taiwan University, Taipei 10617, Taiwan

**Keywords:** cobalt telluride, heteroatoms doping, dual vacancies, biomass derived carbon, carbon fiber, supercapacitors

## Abstract

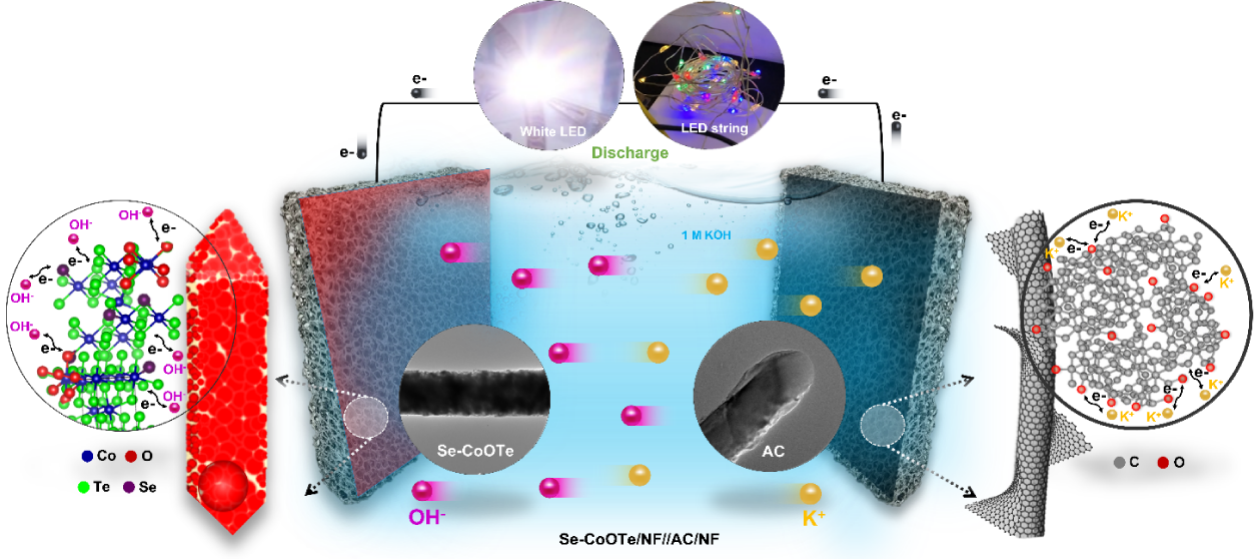

In this work, we demonstrated the synthesis of anions (X = selenium (Se), sulfur (S), and phosphorus (P)) doped cobalt oxytelluride (X-CoOTe) with oxygen and tellurium dual vacancies using hydrothermal methods, followed by selenization, sulfurization, and phosphorization reactions. Especially, the Se-CoOTe-modified nickel foam (Se-CoOTe/NF) electrode delivered a higher specific capacity (752.95 C/g) and an extremely lower charge transfer resistance (0.87 Ω) than S-CoOTe/NF and P-CoOTe/NF due to the higher metallic conductivity of Se. Both oxygen and tellurium vacancies facilitate higher charge transfer conductivity, specific capacity, and stability. On the other hand, banana stem core fiber-derived activated carbon fiber (AC) with exfoliated carbon sheet, cracked surface, and corresponding high surface area boosts the excellent cycle stability up to 4000 cycles with capacitance retention of 100.29%. Thus, the asymmetric device (Se-CoOTe/NF//AC/NF) exhibited an extendable cell voltage (1.55 V), higher energy density (155.6 W h kg^–1^) at a power density (1356.2 W kg^–1^), and generous long-term stability (100% retention up to 10 000 cycles) in a liquid alkaline electrolyte. In the practicability test, the proposed asymmetric device mutually showed an increased operating voltage from 1.55 to 4.65 V for a three-series connection. In a three-series connection, a single white LED and an LED string glowed efficiently. This new finding will be very useful to develop tellurium-based chalcogenides and biowaste-derived carbon for energy storage applications.

## Introduction

Among many different layered nanomaterials, the Co-based metal chalcogenides (CoX_2_, X = S, Se, and Te) have been researched as attractive candidates for electrochemical applications due to various desirable physical and electrochemical properties.^1−5^ Especially for energy storage applications, CoX_2_ exhibits satisfactory properties, such as strong binding energy between Co and X, appropriate channel size in the crystal structure for larger diffusion of ions during charge/discharge reactions, highest surface area per atom facilitates higher specific capacitance, and higher theoretical quantum specific capacitance. In addition, Co*X*_2_ possesses a more enriched density of states near the Fermi level and thus exhibits a higher metallic nature than other first-row transition metal-based chalcogenides.^6−8^ Herein, the metallic conductivity and charge storage performance of CoX_2_ mainly depend on Co 3d electrons in the d orbital with a low spin configuration.^9^ On the other hand, Te has a higher intrinsic electrical conductivity of 2*10^2^ S m^–1^ when compared to Se (1*10^–4^ S m^–1^) and S (5*10^–16^ S m^–1^).^10^ It follows the hypothesis that the lower electronegativity (χ) of chalcogen atoms (Te = 2.1, Se = 2.55, and S = 2.58) is proportional to the degree of covalency and metallic nature.^11^ Although the retention and charge transfer resistance of the CoTe-based mono- and ternary metal chalcogenides are similar to those of S- and Se-based chalcogenides, the specific capacity of the CoTe should be improved further.

Recently, the heterostructure of oxide with metal chalcogenides affords additional active sites and improved cycle stability for electrochemical charge/discharge reactions. Because the introduction of an oxygen atom into the crystal lattice of metal chalcogenides increases the bond length between metal and anion atoms, and thus, enhances the electrical conductivity and active edge sites.^12−14^ For example, Ramulu et al. synthesized the mixed metallic oxysulfide- and oxyphosphide-based electrodes and conducted supercapacitor studies. In this research, metallic oxysulfide and oxyphosphide exhibited higher supercapacitor performances than those of pure metal oxides.^15^ Most research has focused on the mixed bimetallic and trimetallic cations-based Mn-Co oxysulfide,^16^ Ni-Co oxyphosphide,^17^ NiMoOSe,^18^ and CoZnNi oxyphosphide^19^ for energy storage applications.

Just like metal cations, anions (S, Se, and P) doping with metal chalcogenides also affords fantastic engineering strategies for energy storage applications.^20−23^ Mainly, anion doping with metal chalcogenides facilitates the creation of abundant active sites by inducing defects/distortion in the basal plane, decreasing the charge transfer resistance, generating the Fermi energy level with the additional density of states, increasing the quantum capacity, increasing the hydrophilicity, superior long-term stability, and faster ion diffusion for enhancing capacity.^24−29^ Although anion doping shows positive effects, there are no noticeable research studies on anion-doped metal oxychalcogenides for energy storage applications. Carbon nanomaterials with different dimensions have been synthesized and used as an anode materials in energy storage applications. Generally, the properties of each carbon nanomaterial have been categorized based on their dimensional structure.^30−32^ Except for industrial methods (ex: arc discharge and laser ablation), simple synthesis processes and sources are the most searched for carbon materials synthesis. According to the requirement, carbon synthesis from biowaste and, particularly in terms of dimensional structure, is more encouraged due to the simple synthesis procedure, abundance, and low-cost carbon source.^33,34^

**Scheme 1:**
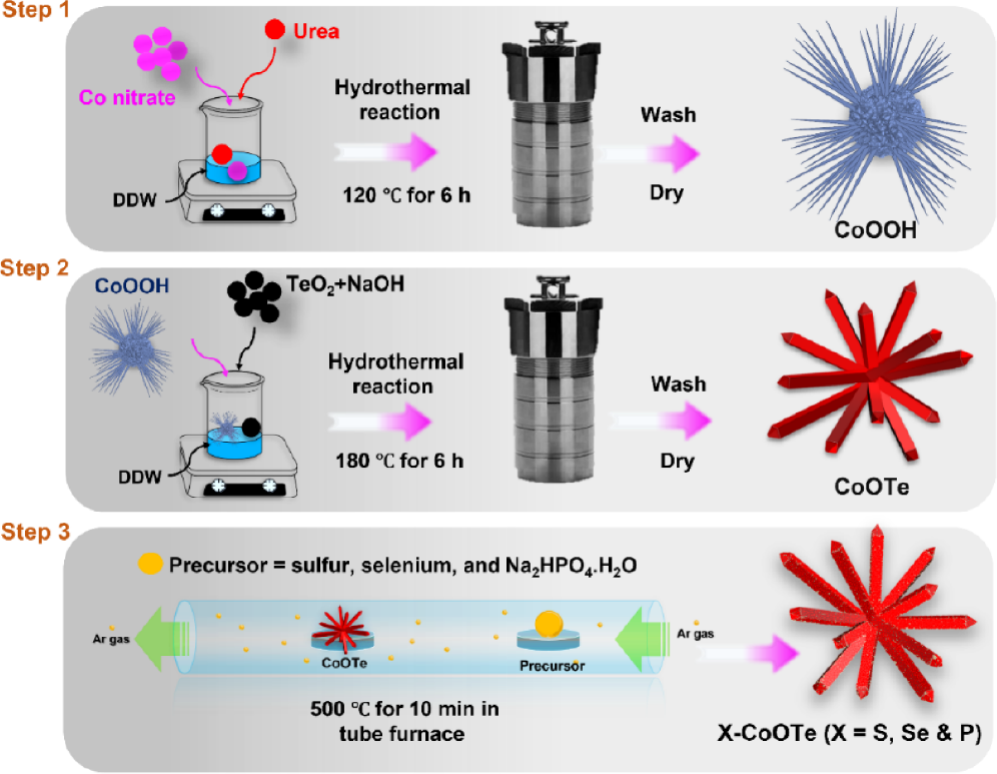
Schematic Representation for the Synthesis of X-CoOTe (X = S, Se, and P).

Therefore, CoOTe was prepared in this study by using the hydrothermal method and subsequently doped with S, Se, and P under sulfurization, selenization, and phosphorization in a tube furnace. On the other hand, AC was prepared from banana stem core fiber by using chemical activation and carbonization methods. The above synthesis procedures for X-CoOTe (X = S, Se, P) and AC are schematically demonstrated in Schemes 1 and S1, respectively. The S-CoOTe, Se-CoOTe, P-CoOTe, and AC-modified NF electrodes were used as the working electrodes in the three-electrode system and their electrochemical properties were studied. Owing to the higher C_s_ and lower *R*_ct_, Se-CoOTe/NF and AC/NF were used as the cathode and anode electrodes in the asymmetric supercapacitor device (Se-CoOTe/NF//AC/NF), respectively. Fortunately, the Se-CoOTe//AC device shows a larger operational potential window of 1.55 V along with a higher energy density/power density and long-term stability. Furthermore, the series connection of three asymmetric devices was tested for commercial applications by lighting up a single white LED and LED string lights with different colors.

## Experimental Section

### Materials and Reagents

Cobalt nitrate hexahydrate, urea, tellurium oxide (TeO_2_), sodium hydroxide (NaOH), potassium hydroxide (KOH), selenium powder, sulfur powder, disodium hydrogen phosphate (Na_2_HPO_4_), 1-methyl-2-pyrrolidinone, polyvinylidene fluoride, carbon black, and carbon nanofiber (CNF, *D* × *L * =100  nm × 20–200 μM, average pore volume = 0.075 cm^3^/g) were purchased from Sigma Aldrich. All chemical reagents were of analytical grade and were used without any further purification.

### Synthesis of X-CoOTe (X = S, Se and P) and AC

To synthesize X-CoOTe (X = S, Se, and P), precursor materials, such as CoOOH and CoOTe, were prepared by using a two-step hydrothermal method. In the first step, cobalt nitrate hexahydrate (0.55 M) was dispersed in 25 mL of DD water and stirred for 10 min. Then, 2.5 M of urea was added to the above solution, and stirring was continued again for 20 min. Herein, urea was used as an attractive precipitation agent for preparing cobalt oxides with unique physical and electrochemical properties. After stirring, the mixture solution was transferred into a stainless steel-covered Teflon autoclave and subjected to the hydrothermal treatment at 120 °C for 6 h. The resultant CoOOH product was washed several times by using EtOH/H_2_O and dried at 60 °C for 12 h. The corresponding reaction mechanism for the above synthesis procedure is given below
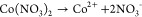
1
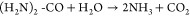
2
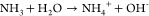
3
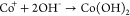
4

From the above eqs 1–3, it can be seen that both Co(NO_3_)_2_ and (H_2_N)_2_-CO dissolve in water and thus release Co^2+^ and OH^–^ ions. In the final step (eq 4), Co(OH)_2_ formed due to the reaction between the Co^2+^ and OH^–^ ions. Herein, Co(OH)_2_ is thermodynamically unstable, and thus it can be convered into the CoOOH product as a result of a hydrothermal reaction. In the second hydrothermal step, 0.25 g of prepared CoOOH was dissolved in 25 mL of DD water and stirred for 10 min. At the same time, TeO_2_ (0.12 M) and NaOH (0.24 M) were dissolved in 25 mL of DD water and added to the above solution of CoOOH. Herein, NaOH was used to dissolve the TeO_2_ precursor. The above mixture was kept in continuous stirring for 30 min and then transferred to anautoclave setup at 180 °C for 6 h. After the hydrothermal process, the obtained CoOTe powder was collected and washed several times by using an EtOH/H_2_O mixture and dried at 60 °C for 12 h. The corresponding reaction mechanism for the CoOTe synthesis procedure is given below
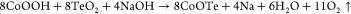
5

In the third synthesis step, the obtained CoOTe powder was subjected to sulfurization, selenization, and phosphorization reactions by using the corresponding dopant precursors, such as sulfur, selenium, and Na_2_HPO_4_, respectively. For the selenization reaction, CoOTe and selenium powder (1:10 wt%) were placed in the tube furnace and a temperature of about 500 °C was applied for 10 min. A similar procedure was followed for both sulfurization and phosphorization reactions. For the synthesis of AC, the banana stem core was collected from a riverside agriculture form in Yonghe, New Taipei City, Taiwan. After that, the banana stem core was washed with DD water and cut into small pieces. Herein, the core fiber can be collected for each cutting of small pieces. In addition, the presence of biofiber between small pieces of the banana core is demonstrated in the photograph (Scheme S1). The collected fibers were washed well using DD water and dried at 80 °C for 48 h. The dried fiber was ground well and subjected to a pre-carbonization reaction at 900 °C (5 °C/min) for 2 h. After the pre-carbonization reaction, the collected pre-carbonized_C was mixed with KOH in the ratio of 1:3 wt% and kept for 24 h. The colloidal mixture of pre-carbonized_C and KOH was again placed in the tube furnace and carbonization was carried out at 900 °C (5 °C/min). Then, the resultant AC was collected and washed with HCl to a solution to become a neutral pH. Finally, the washed AC was dried in a vacuum oven overnight.

### Characterization Techniques

The surface morphology and elemental mapping of prepared Se-CoOTe, S-CoOTe, P-CoOTe, CoOTe, CoOOH, pre-carbonized_C, and AC were analyzed by using the field emission scanning electron microscopy (FE-SEM attached with energy dispersive X-ray (EDX), Nova NanoSEM230, USA) and the transmission electron microscopy (JEOL JEM-2100F instrument JEOL 2100F) techniques. The crystalline properties and electronic states of the synthesized products were confirmed by using the X-ray diffraction technique (XRD, XPERT-3 diffractometer with Cu K_α_ radiation (K= 1.54 Å)) and X-ray photoelectron spectroscopy (XPS, Thermo Scientific Theta Probe, East Grinstead, UK) analyses. The Brunauer–Emmett–Teller (BET) technique (Micrometrics ASAP 2020 M instrument, Norcross, Georgia, USA) was used to analyze the specific surface area and pore size information of the synthesized products. The electrochemical properties of active materials modified with NF were studied by using cyclic voltammetry (CV), and charge–discharge (CD) techniques (CHI440 electrochemical analyzer). Electrochemical impedance spectroscopy (EIS) was carried out by using the IM6ex ZAHNER impedance measurement unit.

The crystallite size of the prepared cathode active materials was calculated by using the Scherrer equation (eq 6)
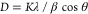
6

where *K* represents the Scherrer constant (0.9), λ is the X-ray wavelength (0.15406 nm), β represents the peak width of the diffraction peak profile at half-maximum height resulting from the small crystallite size in radians, and θ is the Bragg angle.

### Experimental Setup of Three- and Two-Electrode Cells

Before designing the three- and two-electrode systems, the active materials (Se-CoOTe, S-CoOTe, P-CoOTe, CoOTe, CoOOH, pre-carbonized_C, and AC) modified NF should be fabricated. For this, Se-CoOTe (80 wt%), carbon black (15 wt%), and poly(vinylidene fluoride) (5 wt%) were ground with 400 μL of 1-methyl-2-pyrrolidinone by using agate mortar. Then, the 40 μL of resultant mixture was coated on hydrochloric acid-pretreated nickel foam (NF, 1.0 × 1.0 cm^2^) and dried in the vacuum oven at 100 °C for 12 h. All modified electrodes were prepared with 1 mg of mass loading and used for further electrochemical characterizations. The fabricated electrode is represented as a Se-CoOTe/NF. A similar procedure was followed to fabricate S-CoOTe/NF, P-CoOTe/NF, CoOTe/NF, CoOOH/NF, pre-carbonized_C /NF, and AC/NF. In the three-electrode configuration, the modified NF, Pt wire, Hg/HgO (1.0 M KOH), and aqueous 1.0 M KOH were used as the working electrode, auxiliary electrode, reference electrode, and electrolyte, respectively. For the three-electrode system, the CV technique was carried out by applying the potential window (Δ*V*) and scan rate (ν) for the cathode (Δ*V* = −0.4 to 0.6 V and ν = 10 to 50 mV s^–1^) and cathode (Δ*V* = 0 to −0.95 V and ν = 10 to 100 mV s^–1^). The GCD was recorded by applying Δ*V* and current density (*j*) for the cathode (Δ*V* = 0 to 0.5 V and *j* = 2.5 to 6.5 A/g) and anode (Δ*V* = 0 to −0.95 V and *j* = 0.5 to 3 A/g). The EIS was measured in the frequency range from 0.1 Hz to 100 kHz with applied potentials of 0.5 V (cathode) and −0.3 V (anode). In the case of the two-electrode systems, Se-CoOTe/NF and AC/NF are used as the cathode and anode, respectively, whereas 1.0 M KOH was used as the liquid electrolyte. Herein, the expanded Δ*V* was applied in both CV and GCD (Δ*V* = 0 to 1.55 V). In addition, different ν (10 to 50 mV s^–1^) and *j* (1.4 to 3.4 A/g) were applied for CV and GCD experiments, respectively.

The specific capacity (*C*_*s*_) of the modified electrodes is calculated by using the following equation (Eq 7):
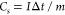
7where *I* represents the applied current density, Δ*t* is denoted as discharge time, and *m* refers to the mass of the active material on the NF substrate.

The equation of charge balance theory can be written as the following equation (Eq 8):
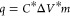
8where *C* represents specific capacitance, Δ*V* is referred to the potential window, and *m* is the mass loading. The above eq 8 can be modified based on *q*^+^ = *q*^–^ as below:
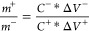
9where *m*^+^ and *m*^–^ represent the mass loading on the positive and negative electrodes, respectively. *C*^–^ and *C*^+^ are denoted as the specific capacitance of the negative and positive electrodes acquired in a three-electrode system, respectively. And, Δ*V*^–^ and Δ*V*^+^ are the applied potential windows for the negative and positive electrodes in a three-electrode system, respectively.

Moreover, the energy density (*E*) and power density (*P*) of the asymmetric Se-CoOTe/NF//AC/NF device were valued by using the following equations (Eqs 10 and 11):
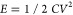
10
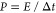
11where *V* is the potential range and Δ*t* denoted the discharge time of the GCD curve.

## Results and Discussion

**Figure 1:**
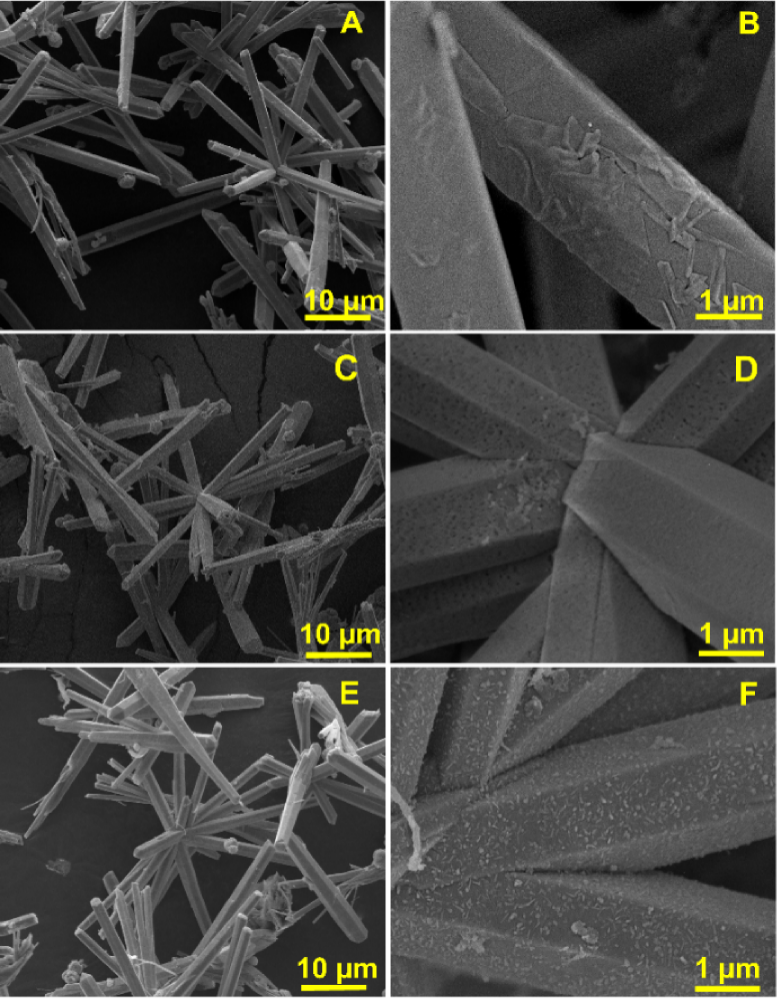
Different magnified FESEM images of (A, B) Se-CoOTe, (C, D) S-CoOTe, and (E, F) P-CoOTe.

First, the surface morphology of source materials, including CoOOH and CoOTe, was studied by using FESEM analysis, as shown in Figures S1 and S2. It can be observed that CoOOH and CoOTe exhibit sea urchin-like and microflower-like structures, respectively. The detailed explanation is provided in the Supporting Information. In the final synthesis process, the different anions Se, S, and P were doped with CoOTe by using the selenization, sulfurization, and phosphorization techniques. The doping of the anions and its effect on the morphology were preliminarily confirmed by using FESEM, as shown in Figure 1. The doping of anions with CoOTe does not show any morphological destruction but induces surface roughness and tip damage in the rod-like structure of Se-CoOTe (Figure 1A,B), S-CoOTe (Figure 1C,D), and P-CoOTe (Figure 1E,F). Herein, Se-CoOTe and S-CoOTe exhibit more surface roughness, porous structure, and tip-edge damage than the P-CoOTe sample. The resultant roughness, porous structure, and tip edge damage make the additional active sites for ion and electron diffusion during the charge/discharge reaction. The successful doping of anions with CoOTe was initially confirmed using EDX analysis. The EDX elemental mapping of Se-CoOTe (Figure S5A–D) shows an equal distribution of Co, O, Te, and Se. Similarly, S-CoOTe (Figure S5E,H) and P-CoOTe (Figure S5I,L) also show the mapping results for the corresponding major elements (S and P) in CoOTe. For further confirmation, the concentration of major elements Co, O, Te, S, Se, and P was calculated and demonstrated in Table S1.

**Figure 2:**
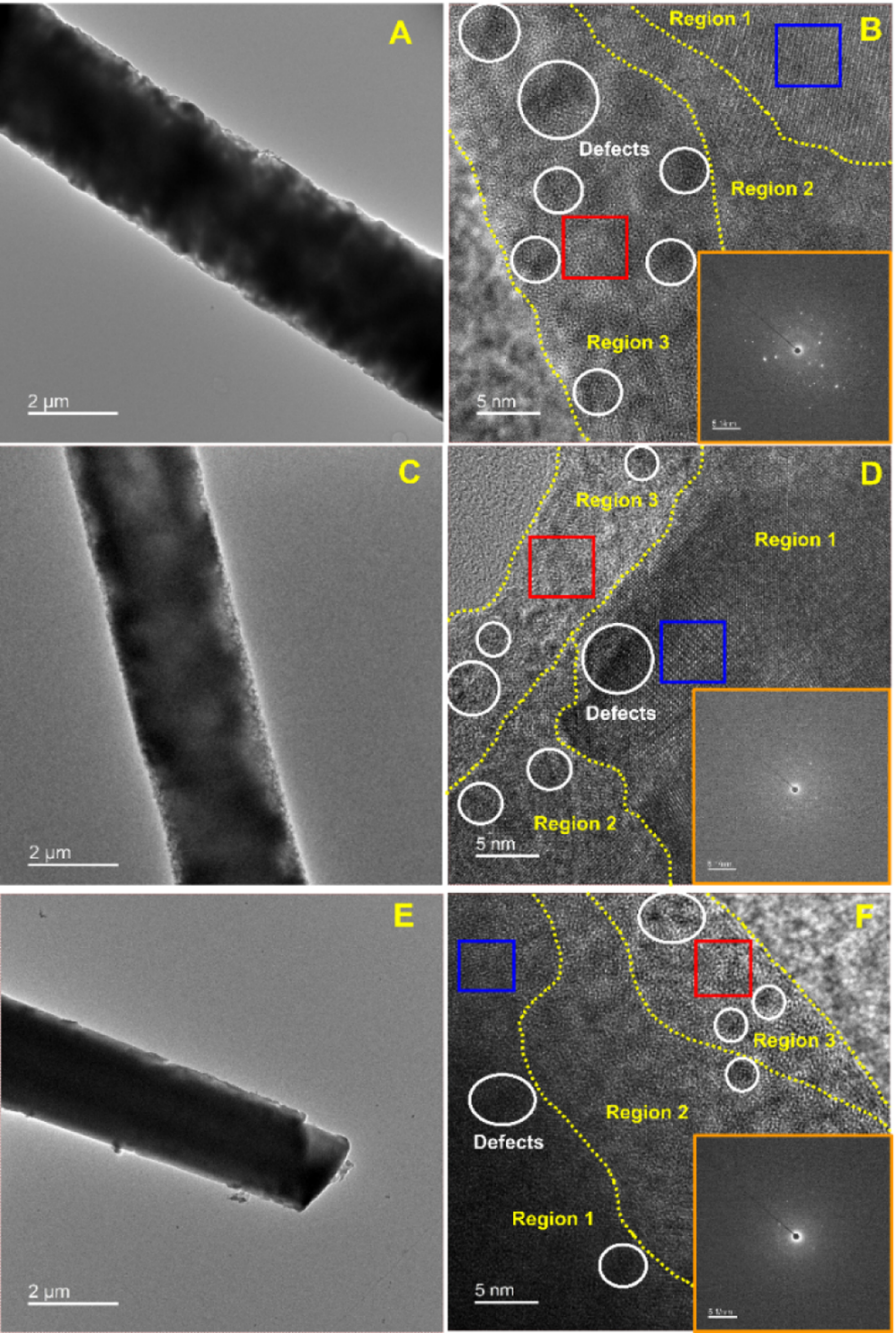
(A, C, and E) TEM and (B, D, and F) HRTEM (inset) SAED patterns of Se-CoOTe, S-CoOTe, and P-CoOTe, respectively.

On the other hand, the EDX quantitative results and line mapping profiles of Se-CoOTe (Figure S6), S-CoOTe (Figure S7), and P-CoOTe (Figure S8) also clearly confirmed the successful doping of anions (Se, S, and P) into the crystal structure of CoOTe. Furthermore, the surface morphology of the prepared samples, heteroatom doping, and its effect on the lattice arrangement were studied and confirmed by using TEM analysis. Figure S9A,B shows the TEM and HRTEM images of the CoOTe rod and its lattice arrangement. The comprehensive explanation is given in Supporting Information. The obtained disordered lattice on the surface of CoOTe can be associated with the formation of oxygen vacancies due to the tellurium ion modulation reaction during the hydrothermal process. The TEM images of Se-CoOTe (Figure 2A), S-CoOTe (Figure 2C), and P-CoOTe (Figure 2E) show similar rod-like structures with surface roughness, porous structure, and tip damage as the result of successful selenization, sulfurization, and phosphorization reactions. The corresponding HRTEM images (Figure 2B,D,F) demonstrate the voids/defects (white circle) at the surface region (Region 3), partial defect at the middle region (Region 2), and nondefect at inner core region (Region 1). The voids/defects formation can be referred to as the Kirkendall effect and nonequilibrium diffusion of anions into the CoOTe crystal lattice.^23,35^ The SAED patterns (Figure 2B,D,F inset) show both bright spots and ring patterns, which indicate that Se, S, and P doping induces the polycrystalline structure. The corresponding IFFT (Figure S10A,B,F,G,K,L) and FFT (inset) patterns from blue and red square areas of HRTEM images also confirmed the presence of both uniform and distorted lattice arrangements in the rod-like structure. From the IFFT pattern of all samples, it can be observed that there is the presence of two different lattice arrangements (uniform lattice and defect lattice). Herein, CoOTe, Se-CoOTe, S-CoOTe, and P-CoOTe exhibit the 0.216, 0.386, 0.120, and 0.281-fold higher defects as compared to the uniform lattice, respectively.

In addition, the heteroatom doping induces different *d*-spacing values due to lattice distortion/defects (Figure S10C–E,H–J,E,H–J,M–O). Herein, the observed lattice disorder confirmed the formation of oxygen and tellurium dual vacancies in the lattice of active materials.^36^ On the other hand, the AC and pre-carbonized_C were characterized by using FESEM, EDX, and TEM analyses. The detailed results (Figures S11–S14) and discussion are provided in Supporting Information.^37^

**Figure 3:**
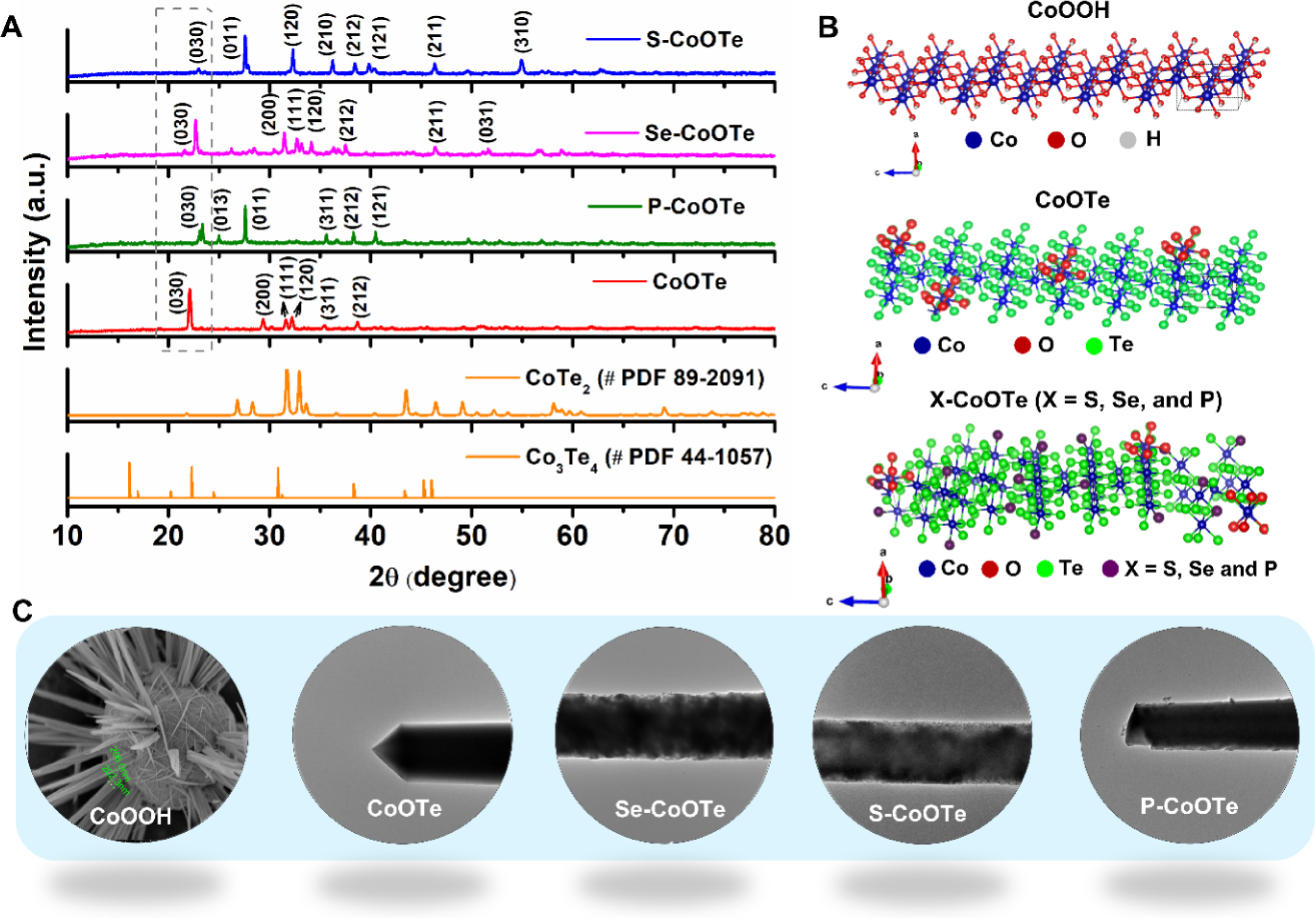
XRD patterns of (A) X-CoOTe (X= S, Se, P) and (B, C) schematic representation for crystal structures of CoOOH, CoOTe, and X-CoOTe (X= S, Se, P) with morphological comparison.

The XRD characterization was performed to understand the crystal nature of the prepared samples. Figure S15A demonstrates the XRD pattern of CoOOH and CoOTe samples with major characteristic lattice planes, which are well matched with the orthorhombic crystal structure of CoOOH (JCPDS no: 26–480), CoTe_2_ (JCPDS no: 89–2091), and Co_3_Te_4_ (JCPDS no: 44–1057) phases. This confirms the formation of CoOOH and CoOTe samples in the first and second hydrothermal processes. After anion doping, the XRD patterns of Se-CoOTe, S-CoOTe, and P-CoOTe (Figure 3A) show the major characteristic peaks with matched lattice planes of orthorhombic crystal structures of CoTe_2_ (JCPDS no: 89–2091) and Co_3_Te_4_ (JCPDS no: 44–1057) phases. In Figure 3A, the dotted line is used to highlight the shift of the major characteristic (030) peak. This is due to the contraction of unit cells in the crystal lattice upon the successful doping of anions (Se, S, and P) atoms with smaller atomic radius than Te.^38^

In addition, the average crystallite sizes of the prepared CoOOH, CoOTe, Se-CoOTe, S-CoOTe, and P-CoOTe samples were calculated to be 17.06, 34.15, 28.20, 25.36, and 25.99 nm, respectively. The detailed calculation is demonstrated in Supporting Information. As per the previously reported study,^39^ the active material with a smaller crystallite size represents more microcrystalline regions and more tortuous migration paths of ions. Meanwhile, the larger crystal size implies longer migration paths of the ions during the charge–discharge process. Therefore, an active material with a moderate crystallite size can deliver a higher discharge capacity. The XRD patterns of pre-carbonized_C and AC are demonstrated in Figure S15B. The detailed results and discussion are provided in Supporting Information.^40^Figure 3B,C demonstrates a schematic representation of the orthorhombic atomic crystal structure and morphological evolution of CoOOH, CoOTe, and X-CoOTe (X = S, Se, and P).

**Figure 4:**
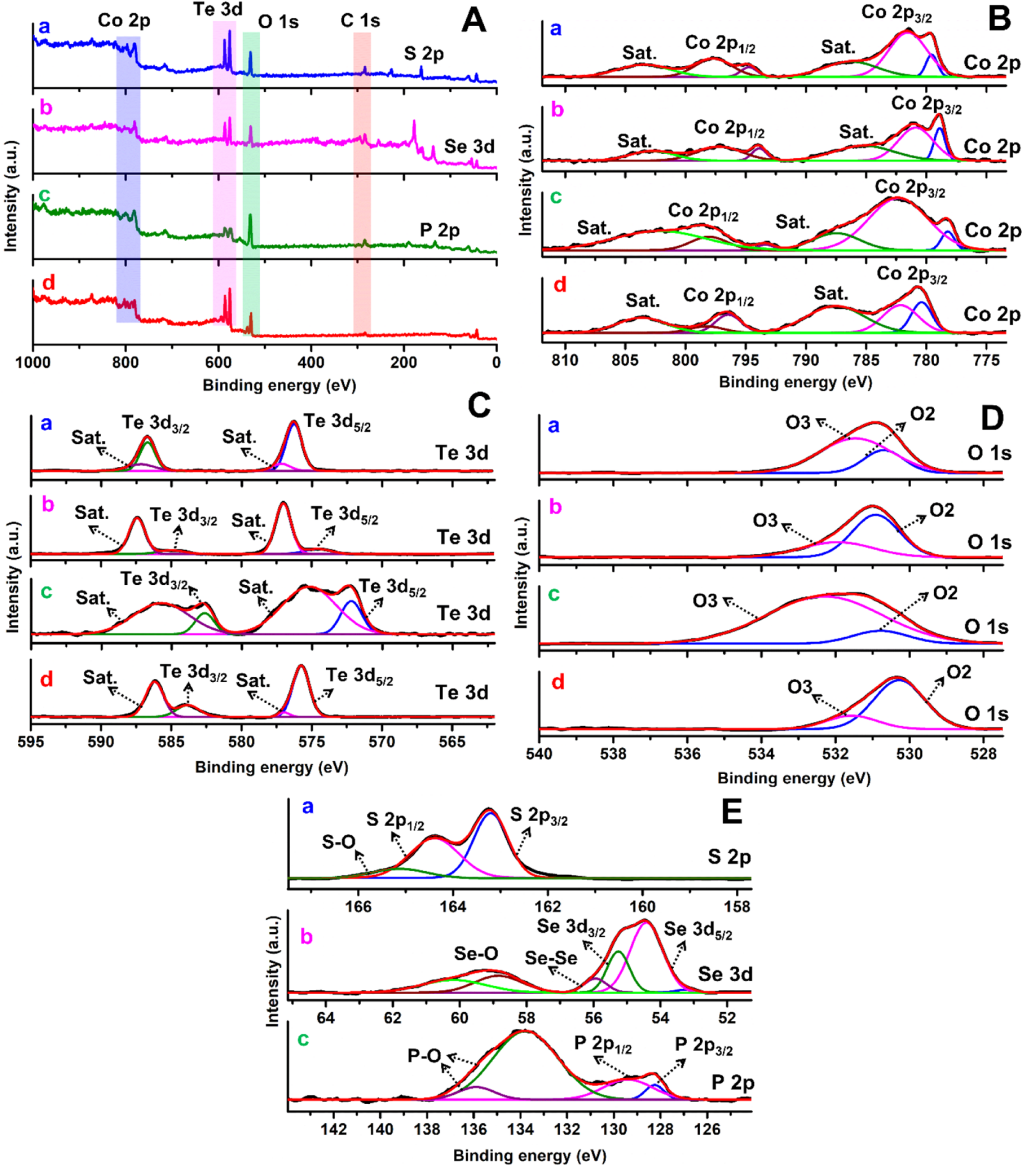
(A) XPS survey spectra and (B–E) high-resolution XPS spectra of (a) S-CoOTe, (b) Se-CoOTe, (c) P-CoOTe, and (d) CoOTe.

The composition and oxidation states of the prepared materials were estimated by using the XPS data. First, the source material CoOOH shows major characteristic peaks for Co and O in the survey spectra (Figure S15C), which suggests the successful formation of the CoOOH sample. The details of the XPS result of CoOOH are described in Supporting Information. In the second step of hydrothermal synthesis process, CoOOH was used as the precursor to prepare CoOTe. Subsequently, S, Se, and P heteroatoms doped CoOTe were synthesized by using sulfurization, selenization, and phosphorization methods, respectively. Figure 4A shows the survey spectra of (a) S-CoOTe, (b) Se-CoOTe, (c) P-CoOTe, and (d) CoOTe, which exhibit common peaks for Co, O, and Te for all samples. Meanwhile, peaks for S, Se, and P can be observed for the corresponding (a) S-CoOTe, (b) Se-CoOTe, and (c) P-CoOTe, respectively. This suggests the successful formation of CoOTe and consequent doping of anions. Heteroatom doping creates different kind of chemical environments for each element in the prepared samples. As seen in Figure 4B, the (a) S-CoOTe, (b) Se-CoOTe, (c) P-CoOTe, and (d) CoOTe show Co 2P spectra with two major characteristic peaks of Co 2p_3/2_ and Co 2p_1/2_ as well as CoOOH.

In (d) CoOTe, the Co^2+^ 2p_3/2_ and Co^3+^ 2p_3/2_ shifted to lower binding energies of 780.34 and 782.08 eV, respectively, due to the reduction tendency of Co and the formation of oxygen vacancies.^36^ The nominal oxidation state of Co in X-CoOTe (X = S, Se, and P) decreased due to the doping of less electronegative S, Se, and P atoms. After heteroatom doping, both Co 2p_3/2_ and Co 2p_1/2_ peaks shifted to lower binding energies due to the formation of Co-X (X = S, Se, and P) binding.^41,42^ It also suggests the formation of tellurium vacancies as a result of anion doping.^36^ A similar effect can be observed in the high-resolution spectra of Te 3d (Figure 4C). Te 3d_3/2_ (575.70 eV) in (d) CoOTe shows a higher binding energy than that of the other spectra. Especially, (b) Se-CoOTe shows the Te 3d spectrum with a suppressed intensity of Te 3d_3/2_ due to the substitution of Te by Se doping, which induces the Te vacancies. The Te vacancies are more favorable for higher diffusion kinetics, thus increasing the capacity, conductivity, and stability.^43^ The spectra of O 1s in (d) CoOTe (Figure 4D) show the lower binding energy of 530.31 eV for O2, which indicates the formation of oxygen vacancies as a result of the tellurium ion modulation reaction in the second step of the hydrothermal process.^18,36^ Herein, the corresponding O2 peak gradually shifts to the positive binding energy due to the doping of S, Se, and P in (a) S-CoOTe, (b) Se-CoOTe, and (c) P-CoOTe. Meanwhile, the O3 in the O 1s spectra shows less intensity in (b) Se-CoOTe due to the lower surface passivation, which can be related to the higher metallic conductivity of Se compared to that of S and P. The higher intensity of the O2 peak in the O 1s spectra of (b) Se-CoOTe confirms the existence of rich oxygen vacancies even after anion doping.

Figure 4E demonstrates the high-resolution XPS spectra for S 2p, Se 3d, and P 2p. In the S 2p spectrum of (a) S-CoOTe, the characteristic peaks at 163.3 164.39, and 165.26 eV for S 2p_3/2_, S 2p_1/2_, and S-O states, respectively.^41^ As seen in the Se 3d spectrum of (b) Se-CoOTe, the characteristic peaks atbinding energies of 54.44 eV, 55.26 eV, 55.95 eV, 58.82 eV, and 59.99 eV for Se 3d_5/2_, Se 3d_3/2_, Se-Se, and Se-O states, respectively.^18^ From P 2p spectra of (c) P-CoOTe, the peaks for P 2p_3/2_, P 2p_1/2_, and p-O can be observed at 128.22 129.45, 133.86, and 135.89 eV, respectively.^42^ These results strongly imply the successful doping of anions into the lattice of CoOTe. On the other hand, the XPS results of pre-carbonized_C and AC are demonstrated in Figure S16A,B. This indicates the successful introduction of oxygen functional groups into the carbon lattice due to the KOH activation process.^44^ The detailed explanation of the XPS results for both carbons is provided in Supporting Information. Thus, the KOH activation of pre-carbonized_C offered beneficial structural effects for higher physical and electrochemical properties.

In addition, both the anode and cathode active materials were analyzed by using the BET technique to understand the specific surface area and pore size distribution. The obtained BET isotherm and BJH pore size profile are demonstrated in Figures S17 and S16C. From this result, it can be observed that Se-CoOTe exhibits a Type-IV isotherm characteristics (mesoporous) with a higher specific surface area (*A*) of 32.92 m^2^/g and a pore diameter (*D*) of 107 Å. A detailed comparison of the BET and BJH pore size profiles for Se-CoOTe, S-CoOTe, and P-CoOTe is explained in Supporting Information. The D and G bands in the Raman spectra provide information on graphitization. In the obtained Raman spectra (Figure S16D), the intensity of the D band is slightly higher for AC (*I*_D_/*I*_G_ = 1) than for pre-carbonized_C (*I*_D_/*I*_G_ = 0.98) owing the higher defective formation as the result of KOH activation process. The detailed explanation is given in Supporting Information. The electrochemical activities of anode and cathode active materials for charge storage performance were studied by using various electrochemical techniques including cyclic voltammetry (CV), galvanostatic charge discharge (GCD), and electrochemical impedance spectroscopy (EIS). First, the CV experiment was performed for cathode active materials modified with NFs, such as Se-CoOTe/NF, S-CoOTe/NF, P-CoOTe/NF, CoOTe/NF, CoOOH/NF, and bare NF.

**Figure 5:**
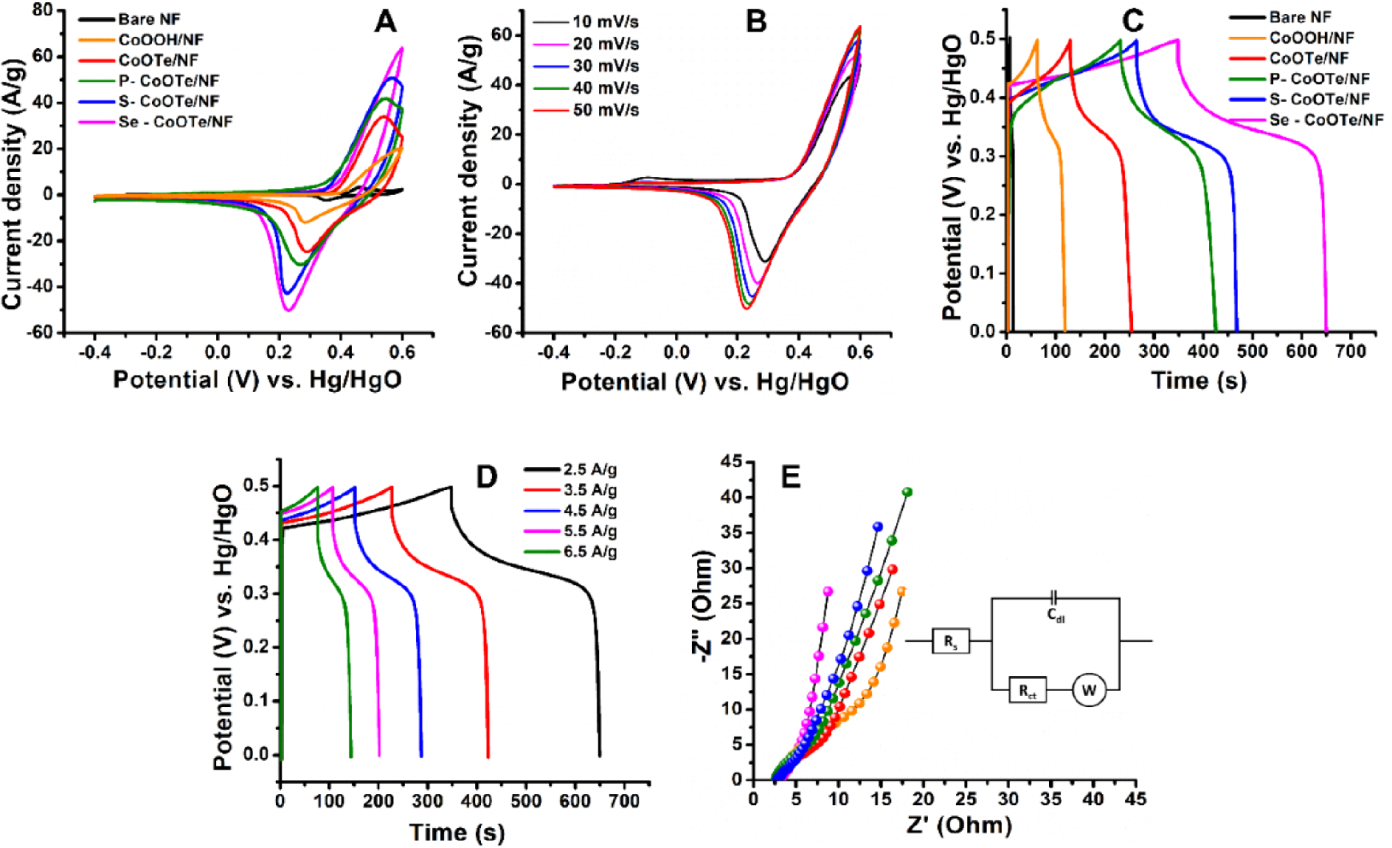
(A) CV curves of different modified electrodes, (B) CV curves of Se-CoOTe/NF for varying scan rates, (C) GCD curve of different modified electrodes, (D) GCD curves of Se-CoOTe/NF for varying current densities, and (E) the Nyquist plot of different modified electrodes (inset) corresponding Randle’s circuit.

The CV experiment was performed at a scan rate of 50 mV s^–1^ and a potential window of −0.4 to 0.6 V. The obtained result is compared and demonstrated in Figure 5A, indicating that the active materials modified NF exhibits the oxidation potential (*E*_pa_ = ∼0.55 V) and reduction potential (*E*_pc_ = ∼0.22 V) responses with higher peak current density in the order of Se-CoOTe/NF > S-CoOTe/NF > P-CoOTe/NF > CoOTe/NF > CoOOH/NF > bare NF. In this result, the redox potential can be associated with the Co^2+^/Co^3+^ redox couple and the higher current density owing to the metallic conductivity of the dopant. This indicates the pseudocapacitive or faradic redox-reaction-based charge storage behavior of the proposed cathode active materials.

In general, the integrated area of the CV curve is directly proportional to the specific capacity of the active materials. As seen in the comparison curves in the CV plot, Se-CoOTe/NF shows the redox behavior with an enlarged CV integrated area of about 0.0209 AV, which is 0.18, 0.25, 0.97, 2.54, and 23.55-fold higher than those of S-CoOTe/NF (0.0176 AV), P-CoOTe/NF (0.0167 AV), CoOTe/NF (0.0106 AV), CoOOH/NF (0.0059 AV), and bare NF (0.000851 AV), respectively. As mentioned in the Introduction section, the heterostructure of CoOTe shows better electrochemical performance than that of CoOOH. Besides, the subsequent doping of anions (Se, S, and P) with CoOTe induced more defects, and thus, facilitated the rich edge active sites, adsorption of reactants, higher electron density, and enhanced intrinsic electronic conductivity for improving the charge storage performances.^20,45−47^ Especially, the Se-CoOTe-modified NF delivered a higher redox reaction due to the higher metallic conductivity of Se as compared to that of S and P. Furthermore, the modified electrodes were tested in the CV experiment by applying different scan rates to understand the reversibility of active materials. Figures 5B and S18A,C,E,G show the CV curves of Se-CoOTe/NF, S-CoOTe/NF, P-CoOTe/NF, CoOTe/NF, and CoOOH/NF for different scan rates from 10 to 50 mV s^–1^. As seen in the results, all modified electrodes show the increased current density with respect to the scan rate. It indicates better reversibility of the modified electrodes.

Figure 5C displays the GCD curve of all proposed electrodes with a fixed potential window (0 to 0.5 V) and current density (2.5 Ag^1–^). From this plot, all modified electrodes delivered the nonsymmetric triangular charge/discharge curve. It also mimics the result of the above CV results in that the modified electrodes show pseudocapacitive charge/discharge behavior. Among these modified electrodes, Se-CoOTe/NF exhibits a larger charge/discharge time and corresponding higher specific capacity of 752.95 C/g. Meanwhile, S-CoOTe/NF, P-CoOTe/NF, CoOTe/NF, and CoOOH/NF electrodes delivered the specific capacity of 512.40 485.15, 312.93, and 140.43 C/g respectively. The specific capacity (*C*_*s*_) of modified electrodes is calculated by using Eq 7.

The GCD experiment for different current densities at Se-CoOTe/NF is demonstrated in Figure 5D. For varying the current density from 2.5 to 6.5 A/g, Se-CoOTe/NF delivered a similar shape to a nonsymmetric charge/discharge curve. It also suggests good reversibility of the modified electrode. In this plot, the charge/discharge time decreases by increasing the current density due to less time for ion diffusion at higher current densities. Figure S18B,D,F,H shows the GCD curves for different current densities at S-CoOTe/NF, P-CoOTe/NF, CoOTe/NF, and CoOOH/NF, respectively, which exhibit a similar shape of nonsymmetric charge/discharge curve as that of Se-CoOTe/NF. Finally, both CV and GCD results confirmed the better electrochemical performance and higher specific capacity of Se-CoOTe/NF, as compared to those of other cathode active materials modified electrodes. It can be related to the presence of rich oxygen and tellurium dual vacancies in Se-CoOTe. The possible redox mechanism of X-CoOTe/NF (X = Se, S, and P) modified electrodes with OH^–^ in the alkaline electrolyte can be written as following equations (12) and (13)^2,3,21^
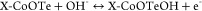
12
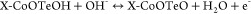
13

The charge transfer resistance is another important criterion to understand the electrochemical activity of the modified electrodes. For this study, Se-CoOTe/NF, S-CoOTe/NF, P-CoOTe/NF, CoOTe/NF, and CoOOH/NF-modified electrodes were tested by using the EIS technique. EIS is a suitable technique to estimate the diffusion efficiency and the quantitative value of charge transfer resistance (*R*_ct_) at the electrode and electrolyte interface. In this experiment, the modified electrodes were tested in 1.0 M KOH electrolyte by applying the fixed frequency range from 0.1 Hz to 100 KHz and a potential of about 0.5 V. The recorded Nyquist plots are shown in Figure 5E, whereas the semicircle in the high-frequency range and Warburg line in the low-frequency region are associated with the *R*_ct_ and ion diffusion efficiency, respectively. It can be observed that Se-CoOTe/NF exhibits lower *R*_ct_ (0.87 Ω) than compare to S-CoOTe/NF (1.67 Ω), P-CoOTe/NF (18.5 Ω), CoOTe/NF (20.99 Ω), and CoOOH/NF (34.5 Ω). Figure 5E (inset) shows the corresponding Randle’s circuit with components of charge transfer resistance (*R*_ct_), electrolyte resistance (*R*_*s*_), Warburg impedance (*Z*_*w*_), and double-layer capacitance (*C*_dl_). It proposes that the interfacial properties of electrodes are related to the aforementioned components. In this plot, the Se-CoOTe/NF exhibits a lower diameter of the semicircle and almost vertical Warburg line than compared to other modified electrodes.

For the anode active materials, CV, GCD, and EIS techniques were performed, and the corresponding results are shown in Figures S19 and S20. From these CV and GCD curves, it can be observed that AC/NF exhibits a larger CV integrate area, lower IR drop, and higher specific capacitance than pre-carbonized_C/NF (see Supporting Information for details). From the plot of current density vs specific capacitance, the capacitance retention was calculated to be 73.67% and 22.00% for AC/NF and pre-carbonized_C/NF, respectively. In contrast, the CE values of AC/NF and pre-carbonized_C/NF are 100.29% and 76.68%, as shown in the current density vs Coulombic efficiency (CE) plot. To understand the charge transfer resistance of AC/NF and pre-carbonized_C/NF, the EIS experiment was performed and demonstrated the results in Figure S20. The better results obtained of AC/NF indicates that the chemical activation process offered a higher surface area, rich defective edge active site, and lower charge transfer resistance (see Supporting Information for details).

Another important characterization is an estimation of the diffusion and capacitive contributions of active materials in total charge storage. By using the power law relationship, the *b* values for CoOOH/NF, CoOTe/NF, Se-CoOTe/NF, S-CoOTe/NF, and P-CoOTe/NF were estimated to be 0.75, 0.87, 1.3, 1.4, and 1.2, respectively (Figure S22A), indicating that the maximum charge storage process follows a complete surface-controlled mechanism (see Supporting Information for details). On the other hand, Dunn and coworkers proposed a method to evaluate the diffusive and capacitive contributions at a specific voltage of a certain scan rate (see Supporting Information for details). It can be observed that the Se-CoOTe/NF exhibits 68.76% capacitive and 31.24% of diffusion. In addition, the capacitive and diffusion percentages were calculated for Se-CoOTe/NF at different scan rates and demonstrated in the bar diagram (Figure S22D). At a lower scan rate (10 mV s^–1^), Se-CoOTe/NF showed the maximum diffusion contribution. This is due to the longer time for diffusion of ions at a lower CV scan rate. While the diffusion percentage gradually decreases by increasing the CV scan rate due to less time for the diffusion of ions. Finally, it implies the maximum contribution of the surface capacitive mechanism for Se-CoOTe/NF at a higher scan rate. By using a similar method, the CV profile of capacitive and diffusion currents and the corresponding bar diagram with quantitative values were calculated for S-CoOTe/NF (Figure S23A,B), P-CoOTe/NF (Figure S23C,D), CoOTe/NF (Figure S23E,F), CoOOH/NF (Figure S23GH), AC/NF (Figure S24A,B), and pre-carbonized_C/NF (Figure S24C,D). The anion doping with CoOTe facilitates the abundant active sites by inducing defects/distortion in the basal plane and thus increase the surface capacitive redox reaction. Among the proposed cathode active materials, Se-CoOTe/NF exhibits a larger surface capacitive contribution at a higher scan rate than compare to S-CoOTe/NF, P-CoOTe/NF, CoOTe/NF, and CoOOH/NF. The better performance of Se-CoOTe/NF can be ascribed to the following reasons: (1) doping with anions in the same group induces slight structural distortion and rich defects with active edge sites for facilitating the electrocatalytic activity.^48^ Herein, S, Se, and Te are the elements 16 in the periodic table, whereas P is the element of the 15 group. Among the same group S and Se, Se exhibits higher intrinsic electrical conductivity than that of S. (2) As per the previously reported study,^39^ the active material with moderate crystallite size can deliver a higher discharge capacity. The average crystallite sizes of the prepared CoOOH, CoOTe, Se-CoOTe, S-CoOTe, and P-CoOTe samples were calculated to be 17.06, 34.15, 28.20, 25.36, and 25.99 nm, respectively. Among these samples, Se-CoOTe exhibits a moderate crystallite size compared to those of other samples.

In the case of anode materials, AC/NF shows 70.49% of capacitive contribution at a lower scan rate than pre-carbonized_C/NF (68%). The higher capacitive contribution of the Se-CoOTe/NF and AC/NF electrodes suggests their surface faradic capacity and double-layer capacitance behaviors, respectively. Besides, the cycle stability of Se-CoOTe/NF and AC/NF electrodes was studied by using GCD, CV, and EIS techniques. For this, Se-CoOTe/NF was performed uisng the GCD technique by applying a fixed current density of 7.5 A/g and a continuous cycle number of about 4000 cycles. The corresponding cycle number vs capacity retention (%) plot is shown in Figure S25A. As seen in the results, the Se-CoOTe/NF shows a capacity retention of 93.16%, whereas the data point for capacity retention and Coulombic efficiency positively fluctuate up to 2000 cycles and after that become stable. It can be related to the initial stabilization of the active material during long-term stability. Figure S25B demonstrates the CV curve of Se-CoOTe/NF before and after 4000 continuous cycles. Both CV curves show an equal integration area of 0.0156. In addition, the GCD curves of the first and last 10 cycles from the total 4000 cycles are demonstrated in Figure S25C,D. In this comparison, the GCD curves of the first and last 10 cycles exhibit a similar nonsymmetric triangle shape. It proved the feasible stability and reversibility of Se-CoOTe/NF. Figure S26A,E shows the CV curves of AC/NF and pre-carbonized_C/NF for 1000 continuous cycles by applying a fixed scan rate of 50 mV s^–1^. The detailed results and discussion are provided in the Supporting Information.

Owing to the higher surface area, lower charge transfer resistance, higher specific capacity, and long-term stability, the Se-CoOTe/NF and AC/NF electrodes were used as cathode and anode electrodes in the asymmetric device (Se-CoOTe/NF//AC/NF), respectively. Before asymmetric device fabrication, the mass loading of active materials on each electrode should be evaluated by using charge balance theory. The equation for charge balance theory is provided in Supporting Information (eqs 8, 9). By using this equation, the mass ratio of Se-CoOTe and AC was found to be 1:4. The calculated mass ratio was used to prepare positive and negative electrodes for further asymmetric device studies.

**Figure 6:**
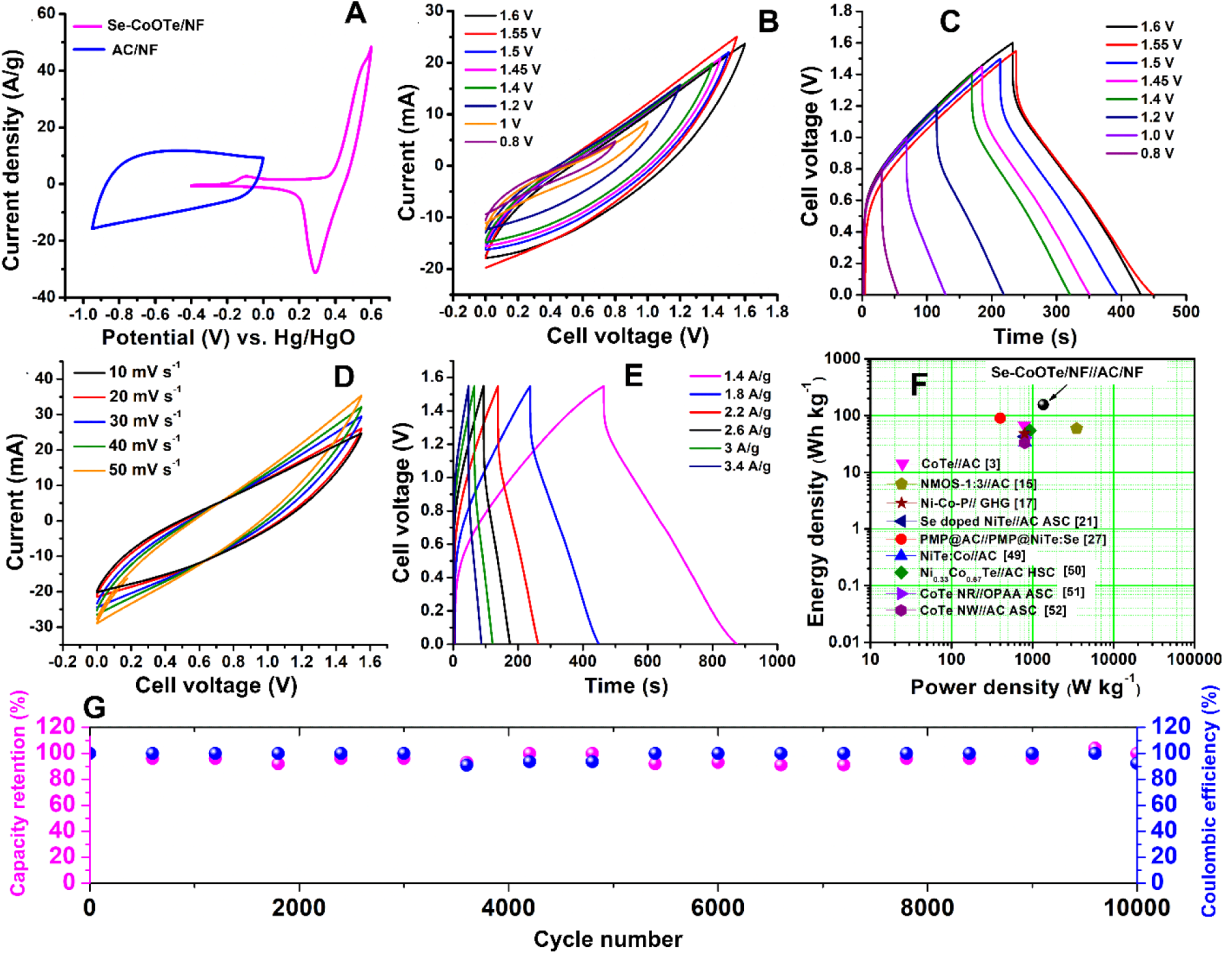
(A) CV profile of the Se-CoOTe/NF- and AC/NF-modified electrodes, (B) CV and (C) GCD curve of the Se-CoOTe/NF//AC/NF asymmetric device with different potential windows. (D) CV curve for different scan rates, and (E) GCD curve for varying current density at Se-CoOTe/NF//AC/NF (potential window; 0 to 1.55 V). (F) Ragone plot,^3,15,17,21,27,49−52^ and (G) long-term stability and Coulombic efficiency of asymmetric supercapacitor device.

For potential window comparison, the CV curves of Se-CoOTe/NF and AC/NF electrodes were recorded in a three-electrode system as shown in Figure 6A. As seen in the comparison curves, the Se-CoOTe/NF- and AC/NF-based asymmetric devices can work in an expanded potential window over > 1.2 V. Therefore, different wide potential windows from 0 to 0.8, 1, 1.2, 1.4, 1.45, 1.5, 1.55, and 1.6 V were applied to a single Se-CoOTe/NF//AC/NF device and recorded the CV and GCD curves, as shown in Figure 6B,C. From this result, It can be seen that the potential window of the proposed asymmetric device can be expanded to 1.6 V. The CV integrate area and GCD discharge time gradually increase by increasing the potential window from 0.8 to 1.55 V. Thus, 1.55 V was fixed as an optimized potential window owing to the maximum discharge time compared to 1.6 V. Therefore, both CV and GCD curves were recorded by applying the optimized potential window (0 to 1.55 V) with different scan rates (10, 20, 30, 40, and 50 mV s^–1^) and current densities (1.4, 1.8, 2.2, 2.6, 3, and 3.4 A/g), as shown in Figure 6D,E. For increasing scan rate and current density, the asymmetric device exhibited an increased CV integrate area and decreased charge–discharge time with similar shapes, respectively. However, the asymmetric device delivered the CV curve with a slanting shape, which indicates the resistive nature of the device. The internal resistance of the asymmetric device depends upon many factors such as electrode surface area, distance between the electrodes, and operating temperature. The high surface area of the active material, less distance between the electrodes, and optimized operating temperature are favorable for obtaining the ideal flat CV curve. The distance between the anode and cathode electrodes in a fabricated asymmetric device is slightly higher (∼1.5 cm). The longer distance between the two electrodes can induce a negative effect for ion transport.^53^ This can be one of the factors for the resistive nature of CV curves of an asymmetric devices.

From the GCD result, the specific capacities of the asymmetric device were calculated to be 722.90, 472.79, 349.69, 261.62, 223.12, and 176.33 C/g for 1.4, 1.8, 2.2, 2.6, 3, and 3.4 A/g, respectively. Moreover, the energy density (*E*) and power density (*P)* of the asymmetric Se-CoOTe/NF//AC/NF device were valued by using the eqs 10, 11. By using the equations, the maximum *E* and *P* of Se-CoOTe/NF//AC/NF were calculated to be 155.6 W h kg^–1^ and 1356.2 W kg^–1^, respectively. Furthermore, the obtained *E* and *P* of the proposed device were compared with those of other previously published works, as shown in the Ragone plot (Figure 6F). From this comparison, it can be concluded that Se-CoOTe/NF//AC/NF exhibited the generous *E* than previously published mono- and bimetallic telluride-based chalcogenides and activated carbon-based asymmetric devices. In addition, Figure 6G confirms 100% higher stability and Coulombic efficiency of the proposed Se-CoOTe/NF//AC/NF device up to 10000 cycles. This study strongly proposes the superior long-term stability of Se-CoOTe/NF//AC/NF. In fact, the GCD curves for the first and last 10 cycles of the Se-CoOTe/NF//AC/NF device (Figure S29) retain a similar shape due to the better reversibility. Besides, the post-stability study of both Se-CoOTe/NF and AC/NF electrodes was performed by using the SEM technique. The corresponding SEM images of both electrodes before and after long-term stability are demonstrated in Figure S30A–D.

**Figure 7:**
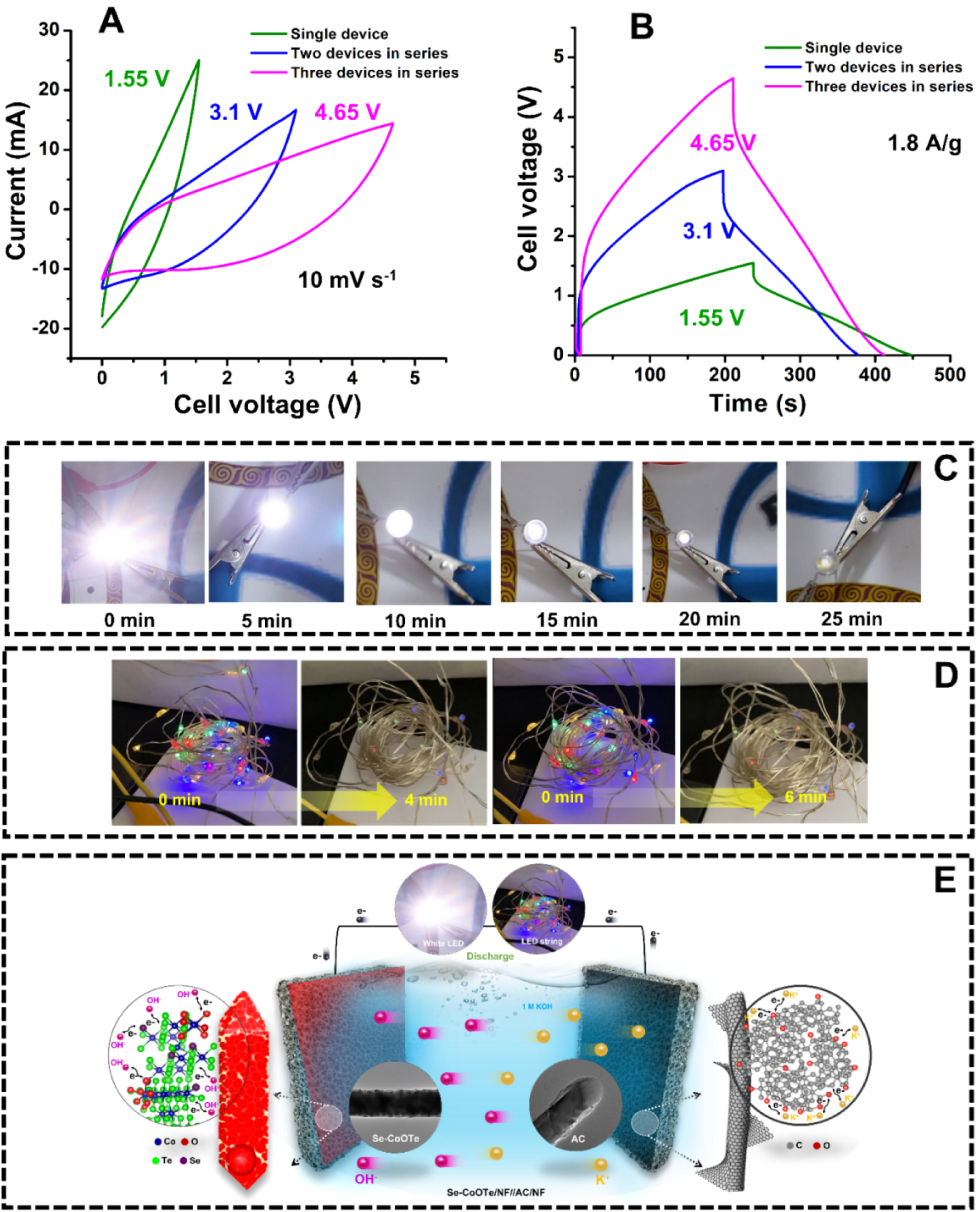
(A) CV curves and (B) GCD curves of single-, two-, and three-Se-CoOTe/NF//AC/NF asymmetric devices in series connection with different potential windows. (C) Photographical images of white LED light testing at different time intervals. (D) Photographical images for LED light string glow testing. (E) Schematic representation of the proposed asymmetric device.

From these images, it can be seen that the rod-like structure of Se-CoOTe slightly split; meanwhile, the fiberlike structure of AC broke due to the long-time charge–discharge of ions. Noticeably, the small change in the morphology of both active materials causes only less fluctuation in the capacity retention and Coulombic efficiency. Finally, Table 1 validates the resultant specific capacity, stability retention, energy density, and power density of the proposed asymmetric device with previously published literature. The practical application of the proposed asymmetric device was experimentally tested by designing the single-, two,- and three-asymmetric devices in series connection. After that, the three-series connection was tested to light up a single white LED (size: 5 mm and voltage: 3 V) and LED string (length: 5 m, number of LED: 50, voltage: 4.5 V, wire: copper, and colors: green, blue, red, and yellow). First, the CV and GCD experiments were executed to record the extended voltage of single-, two-, and three-series asymmetric devices. Figure 7A,B shows the CV and GCD curves of single-, two-, and three-series asymmetric devices with achieved extended voltages of 1.55 3.1, and 4.65 V, respectively. It strongly confirmed the mutual enhancement of the voltage window with the number of devices in a series connection. Thus, further enhancement of the voltage window by increasing the number of devices in series connection can be expected and used for various practical applications. The photographic images of the glow-white LED are demonstrated in Figure 7C. For LED glow, the three-series asymmetric device was charged to 4.65 V by applying a current density of 15 mA. Then, the positive and negative terminals of the asymmetric device were disconnected from the power source and connected to the LED.

**Table 1:** Comparison of Obtained Electrochemical Results of the Proposed Asymmetric Device with Previously Published Works

electrode	substrate	electrolyte	current density (A g^–1^)	Δ*V* (V)	*C*_*s*_ (C g^–1^)	*E* (W h kg^–1^)	*P* (W kg^–1^)	retention (%)	ref.
CoTe//AC	CF	3 M KOH	1	1.6	107.68	23.5	793.5	2000/85	(3)
NiMoOS//AC	NF	2 M KOH	1	1.5	287.7	58.9	3502	10000/90.6	(15)
Se-NiTe//AC	NF	3 M KOH	1	1.6	191.84	42.7	800	10000/76.4	(21)
NiTe:Co//AC	NF	3 M KOH	1	1.6	165.44	36.4	24.4	1000/90	(49)
CoTe//AC	NF	3 M KOH	1	1.66	183	32.9	800.27	5000/76.9	(51)
MnO_x_S_2-x_	NF	1 M Na_2_SO_4_	1	0.8	214.78	-	-	1000/75	(54)
(CoNi)OxSy//AC	NF	2 M NaOH	0.5	0.5	355.2	27	136.9	8000/84.0	(55)
Fe-VO-S//AC	NF	0.5 M Na_2_SO_4_	3	0.8	93.6	9.3	2200	4000/92	(56)
Se-CoOTe//AC	NF	1 M KOH	1.4	1.55	722.90	155.6	1356.2	10000/100	this work

Therefore, the LED started to glow from 0 s to 25 min, whereas the photographic images of LED glowing for each 5 min are demonstrated evidently. In the case of the LED string, the three-series asymmetric device was charged to 4.65 V by applying a current density of 10 mA. Figure 7D shows the photographic images of the glow LED string, herein two glow conditions were applied, including continuous ON condition and ON/OFF (bling) condition. In the continuous ON condition, the LED string continuously glows up from 0 to 4 min. Meanwhile, the LED string in ON/OFF condition glows from 0 to 6 min. For understanding the fabrication of the proposed asymmetric device, the simple schematic representation of the device is demonstrated in Figure 7E. This above experimental study confirmed the excellent practicability of the Se-CoOTe/NF//AC/NF device.

## Conclusions

In summary, X-CoOTe (X = Se, S, P) active materials with oxygen and tellurium dual vacancies were successfully prepared by using two-step hydrothermal and consequent selenization, sulfurization, and phosphorization methods. Additionally, AC was successfully derived from banana stem core fiber by using carbonization and KOH chemical activation methods. It is evidenced from TEM analysis that Se, S, and P doping induces lattice distortion/defects in the crystal structure of CoOTe. As a result of XPS analysis, oxygen and tellurium vacancies were successfully formed in the second hydrothermal and doping processes, which are additionally improving the charge storage performances.^43^ On the other hand, AC was observed with perfect fiber morphology with exfoliated graphene sheets with thickness from ∼ 45.6 to 46.1 nm and cracked texture. Owing to the defective lattice and thus corresponding rich active sites, the anion-doped X-CoOTe (X = S, Se, and P) delivered enhanced electrochemical performances. In the positive potential window, Se-CoOTe-modified NF exhibits a lower *R*_ct_, higher diffusion efficiency, and higher specific capacity than S-CoOTe/NF and P-CoOTe/NF. It can be related to the higher metallic conductivity of Se than S and P,^10,57^ Due to the higher specific surface area, AC/NF delivered lower *R*_ct_, higher specific capacitance, and longer cycle stability in the negative potential window than pre-carbonized_C/NF. When designing the asymmetric device by using Se-CoOTe/NF as the cathode and AC/NF as the anode, it exhibited a larger potential window (1.55 V) along with higher power density/energy density and long-term stability. Fortunately, the three-series connection of the Se-CoOTe/NF//AC/NF device mutually delivered the extendable cell potential up to 4.65 V. Thus, the proposed series connection showed excellent practicability in light glowing tests with a single white LED and an LED string. This present work significantly proposed the effects and advantages of anion doping with metal oxytelluride. It can be an innovative strategy to exploit a new type of telluride-based chalcogenides. It is hoped that this proposed biowaste will become a superior choice to prepare the carbon with a defined dimensional structure for energy storage applications.

## Supporting Information Available

The SEM, EDX, and mapping result of CoOOH, CoOTe, Se-CoOTe, S-CoOTe, P-CoOTe, pre-carbonized_C, and AC. The TEM image of CoOTe. The FFT and IFFT results of Se-CoOTe, S-CoOTe, and P-CoOTe. The XRD patterns of CoOOH, CoOTe, and pre-carbonized_C and AC. The XPS and BET results of CoOOH, pre-carbonized_C, and AC. Raman spectra of pre-carbonized_C and AC. The CV and GCD curves of CoOOH/NF, CoOTe/NF, S-CoOTe/NF, and P-CoOTe/NF with different scan rates and current densities. EIS curves of pre-carbonized_C/NF and AC/NF. The CV curves for the diffusion and capacitive percentage of CoOOH/NF, CoOTe/NF, S-CoOTe/NF, P-CoOTe/NF, pre-carbonized_C/NF, and AC/NF. Cycling stability results of Se-CoOTe/NF, pre-carbonized_C/NF, and AC/NF. Corresponding results and discussions. Post-stability study of Se-CoOTe/NF and AC/NF (PDF)
