# Supplementary material for: X-CoOTe (X = S, Se, and P) with Oxygen/Tellurium Dual Vacancies and Banana Stem Fiber-Derived Carbon Fiber as Battery-Type Cathode and Anode Materials for Asymmetric Supercapacitor
Source: ACS Appl Mater Interfaces. 2024 Apr 2;16(15):18754–67. doi: 10.1021/acsami.3c18205 (PMC11040578; doi:10.1021/acsami.3c18205)
Supplement: Supplementary file 1 — am3c18205_si_001.pdf [file am3c18205_si_001.pdf]

## Supporting Information

# X-CoOTe (X = S, Se, and P) with Oxygen/Tellurium Dual Vacancies and Banana Stem Fiber Derived Carbon Fiber as Battery-Type Cathode and Anode Materials for Asymmetric Supercapacitor

**Mani Sakthivel[a,b], Kuo-Chuan Ho[a,b,c*]**

[a]*Department of Chemical Engineering, National Taiwan University, Taipei 10617, Taiwan*

[b]*Advanced Research Center for Green Materials Science and Technology, National Taiwan University, Taipei 10617, Taiwan*

[c]*Institute of Polymer Science and Engineering, National Taiwan University, Taipei 10617, Taiwan*

**Corresponding author: Tel.: +886–2–2366–0739; Fax: +886–2–2362–3040*

*E–mail: kcho@ntu.edu.tw*

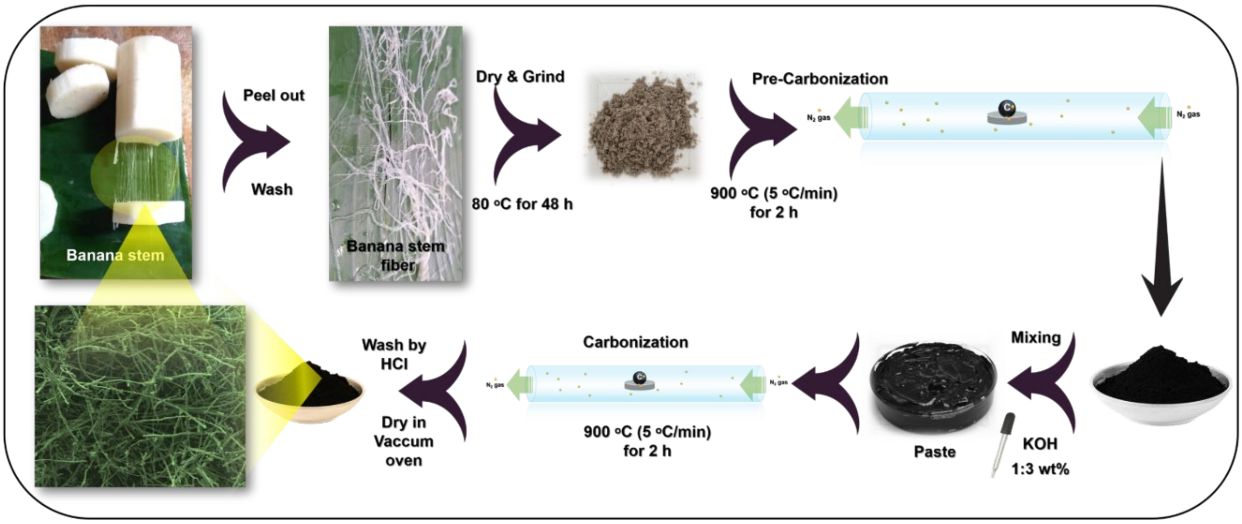


**Scheme S1.** Schematic representation for the synthesis of AC.

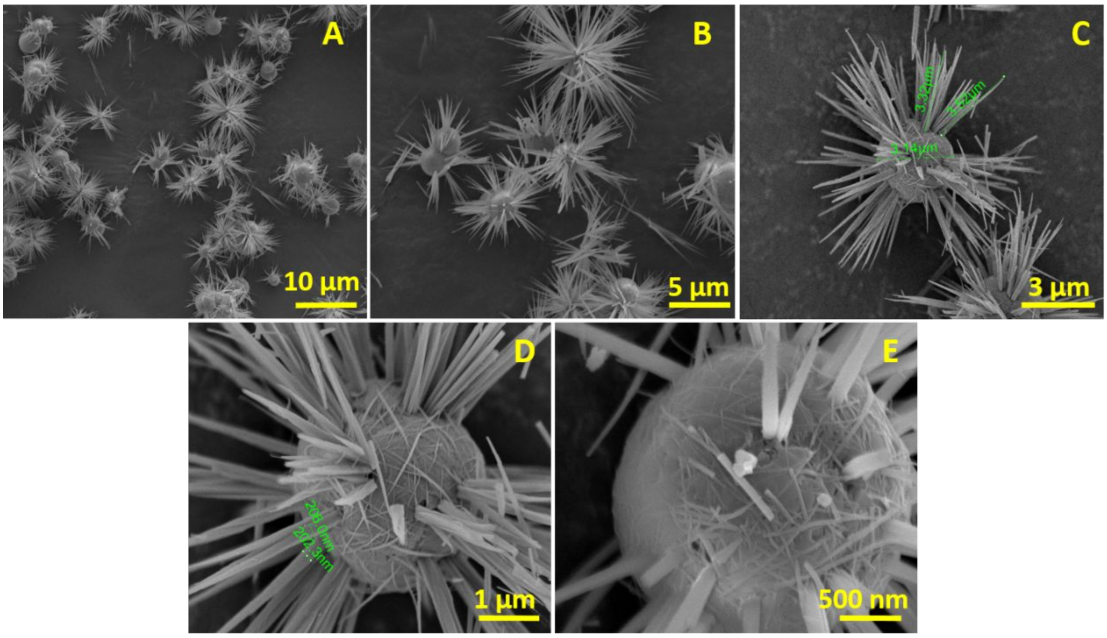


**Figure S1.** (A-E) Different magnified FESEM images of CoOOH.

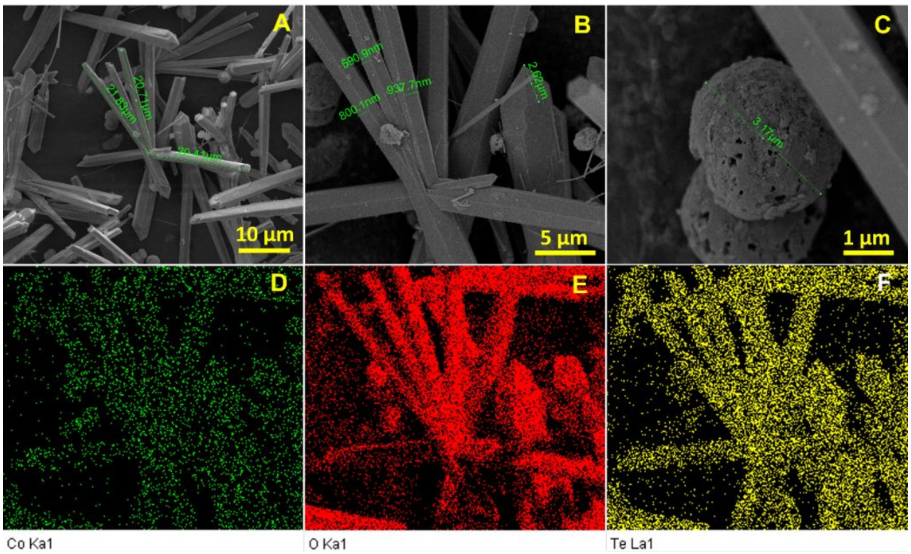


**Figure S2.** (A-C) FESEM images and (D-F) elemental mapping of CoOTe.

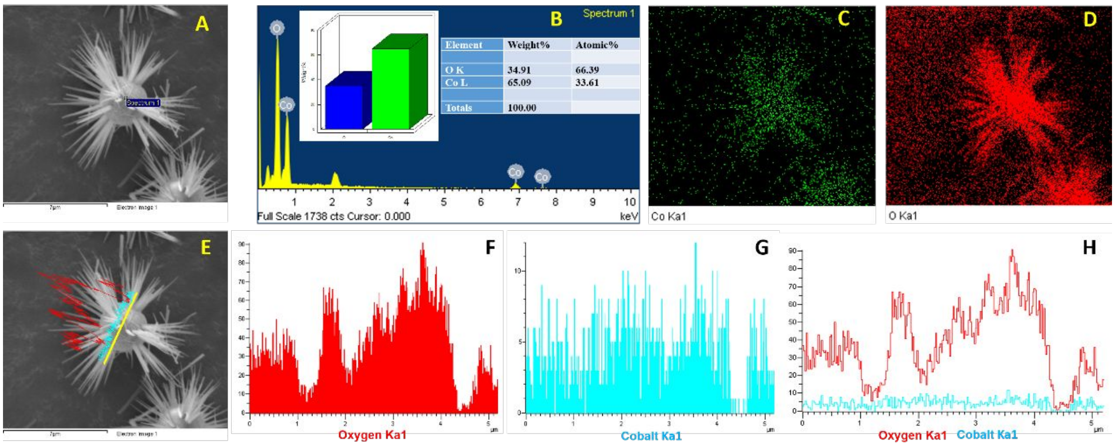


**Figure S3.** (A) FESEM image, (B) EDX spectra and corresponding quantitative result, (C, D) elemental mapping, (E) FESEM image with line mapping data and (F-H) corresponding line mapping spectra of CoOOH.

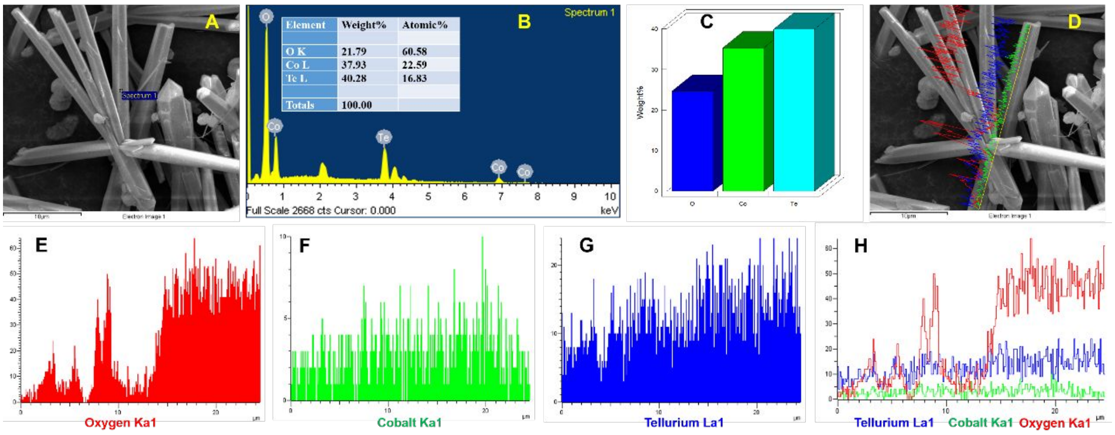


**Figure S4.** (A) FESEM image, (B, C) EDX spectra and corresponding quantitative result, (D) FESEM image with line mapping data, and (E-H) corresponding Line mapping spectra of CoOTe.

As the result of first step hydrothermal process, CoOOH shows the sea urchin-like structure (Figure S1 (A, B)). In the resultant urchin-like structure, the length of the needle (~ 3.52 μm and 3.32 μm), the thickness of the needle (~ 202.3 nm and 206.0 nm), and the diameter of the sphere (~ 3.14 μm) were measured as shown in Figure S1 (C, D). Moreover, Figure S1E shows the growth of the needle on the surface of the sphere, which is also made up of a bundle of ribbon-like structures. Due to the effect of NaOH in the second step hydrothermal process, CoOTe shows the micro flower-like structure made by the convergent of the hexagonal rods with cone-like edges. It can be seen that all the hexagonal rods are grown from a single center and exhibit micro flower-like morphology (Figure S2). The hexagonal rod consisting different geometry sizes including lengths in the range from 20.41 to 21.83 μm (Figure S2A), side wall width from 590.9 to 937.7 nm, and height of cone-like tip ~ 2.62 μm (Figure S2B). According to the previous literature[1, 2], the NaOH induced the growth in the side

wall of the rod only, but no changes in the primary spherical core. Therefore, the spherical structure in CoOTe sample (Figure S2C) also shows a similar diameter as that of the CoOOH (Figure S1C). The preliminary FESEM image and EDX spectra with the quantitative result (Figure S3 (A, B)), mapping (Figure S3 (C, D)), and line profile mapping (Figure S3 (E-H)) also confirmed the formation of CoOOH urchin-like structure with equal distribution of main constituent elements (Co and O). Furthermore, the EDX elemental mapping (Figure S2 (D-F)), the quantitative results (Figure S4 (A-C)), and the line mapping profile (Figure S4 (D-H)) confirmed the equal distribution of Co, O, and Te elements over the surface and thickness of the CoOTe rod-like structure.

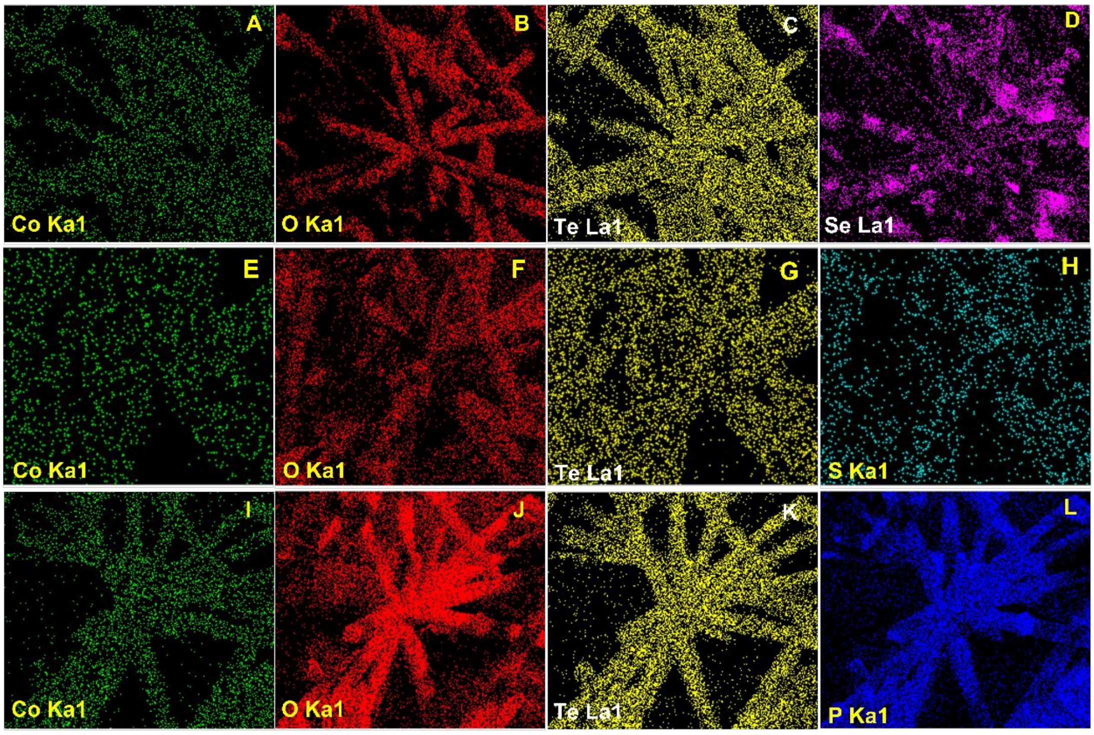


**Figure S5.** EDX elemental mapping result of (A-D) Se-CoOTe, (E-H) S-CoOTe, and (I-L) P-CoOTe.

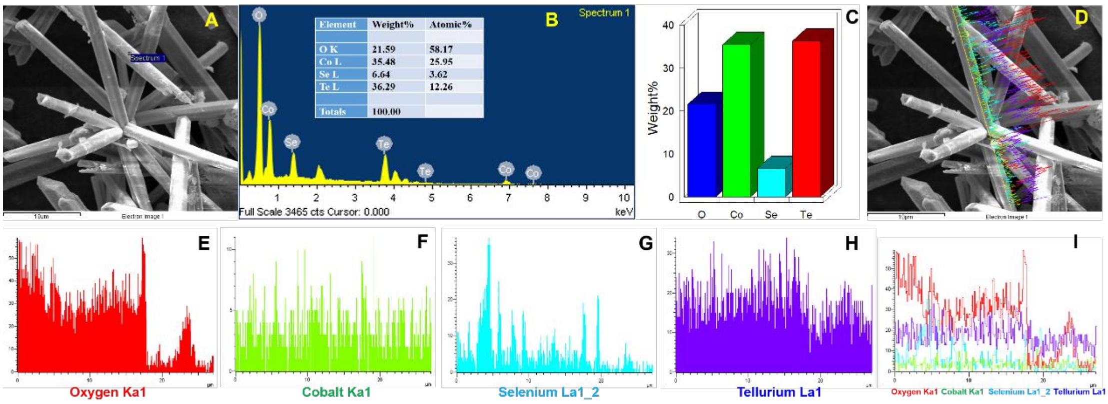


**Figure S6.** (A) FESEM image, (B, C) EDX spectra and corresponding quantitative result, (D) FESEM image with line mapping spectra, and (E-I) corresponding Line mapping spectra of Se-CoOTe.

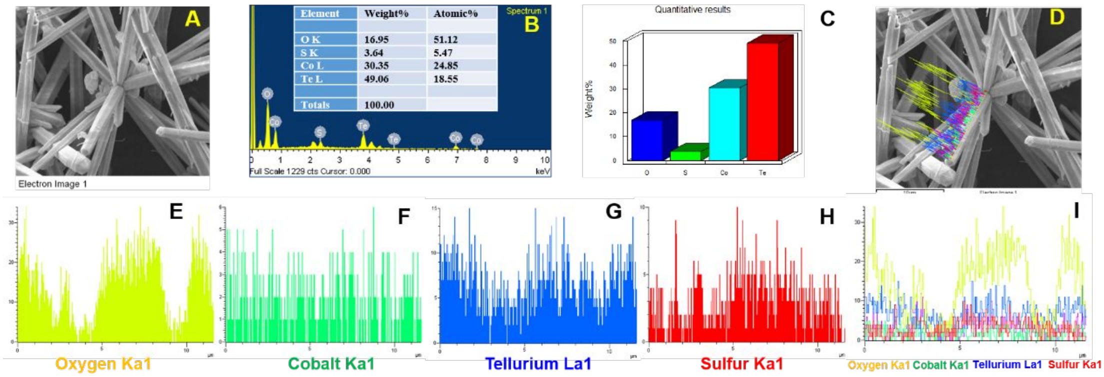


**Figure S7.** (A) FESEM image, (B, C) EDX spectra and corresponding quantitative result, (D) FESEM image with line mapping spectra, and (E-I) corresponding Line mapping spectra of S-CoOTe.

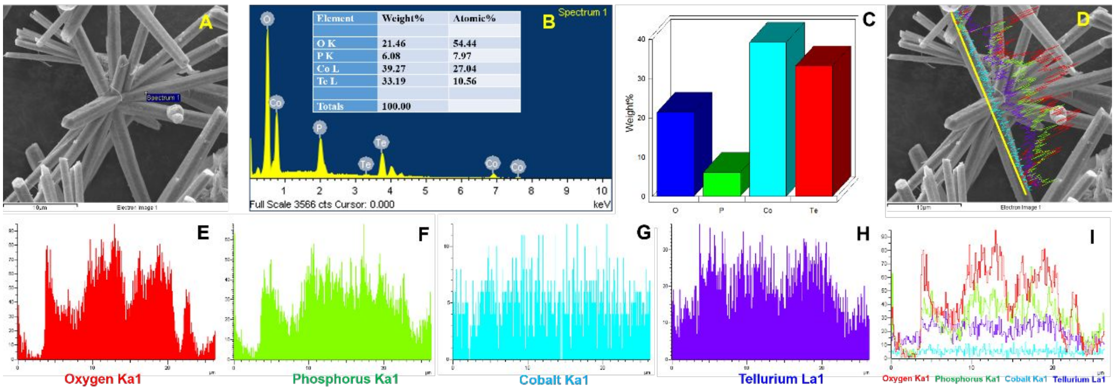


**Figure S8.** (A) FESEM image, (B, C) EDX spectra and corresponding quantitative result, (D) FESEM image with line mapping spectra, and (E-I) corresponding Line mapping spectra of P-CoOTe.

**Table S1.** Concentration of major elements in the prepared samples.

| Samples | Concentration of elements (M) | | | | | |
|---|---|---|---|---|---|---|
| | Co | O | Te | Se | S | P |
| CoOTe | 3.83 | 37.86 | 1.31 | - | - | - |
| Se-CoOTe | 3.89 | 36.36 | 0.96 | 0.45 | - | - |
| S-CoOTe | 4.21 | 31.95 | 1.45 | - | 1.70 | - |
| P-CoOTe | 4.58 | 34.03 | 0.82 | - | - | 2.57 |

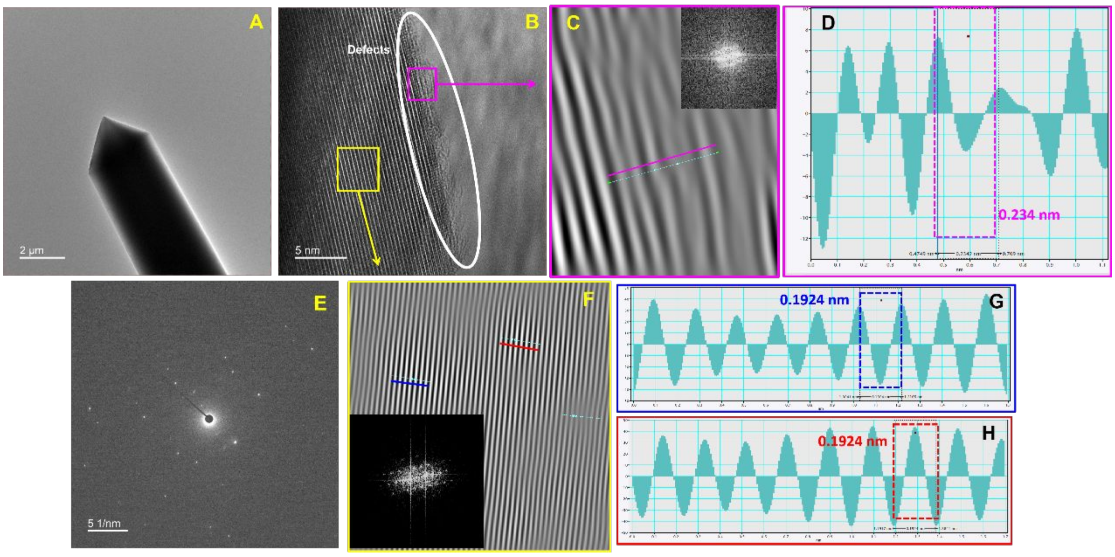


**Figure S9.** (A) TEM, (B) HRTEM, (C, F) IFFT pattern, (insert) FFT pattern, (E) SAED pattern, and (D, G, and H) d spacing line profile of CoOTe.

By using Gatan Digital Microscopy software, the IFFT (Figure S9 (C, F)), FFT (insert), and line profile for d-spacing (Figure S9 (D, G, and H)) of CoOTe were derived from the corresponding HRTEM result (Figure S9B). From the HRTEM, IFFT, and FFT results, it can be observed that the CoOTe rod shows lattice distortion on its surface (white ellipsoid). Moreover, the crystal nature of the inner core of CoOTe rod was identified by observing the regular arrangement of bright spots in the SAED pattern (Figure S9E). The line profile for d-spacing (Figure S9 (D, G, and H)) of CoOTe in different locations shows dissimilar d-spacing values.

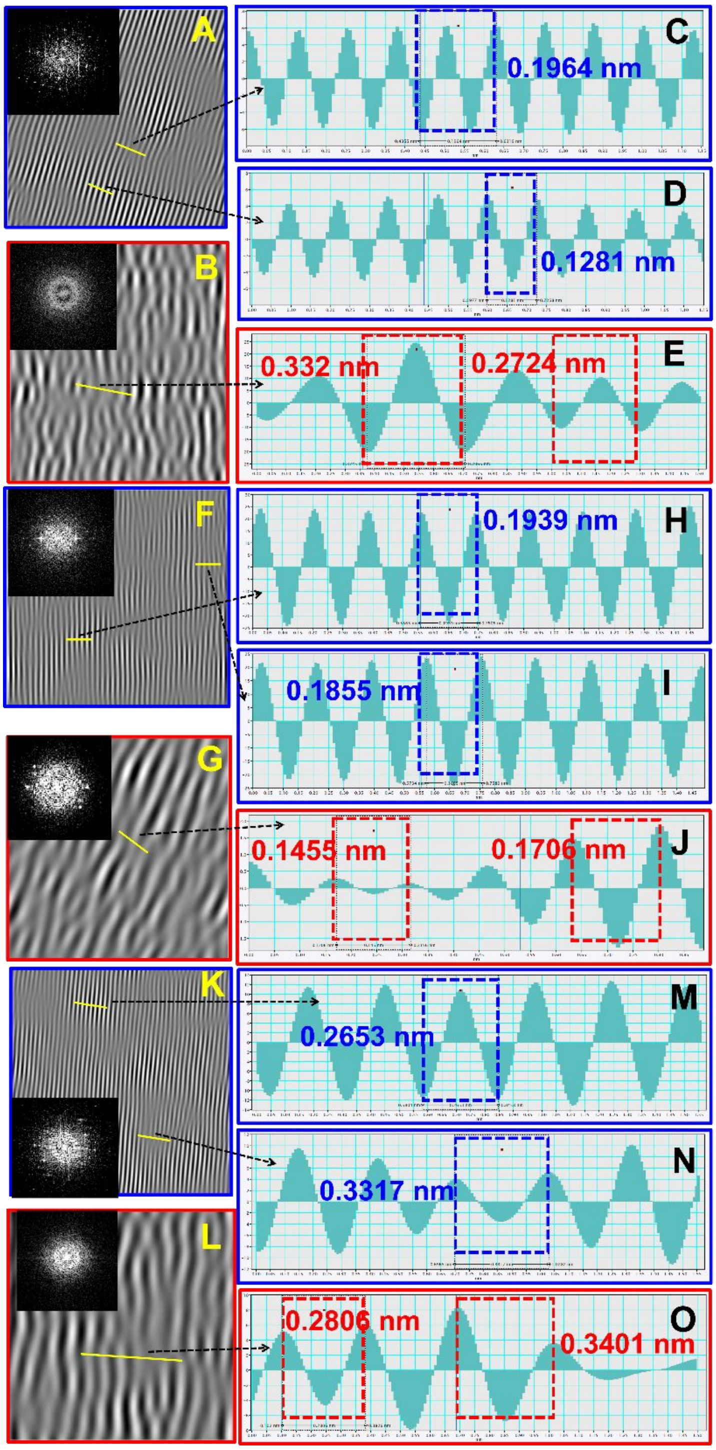


**Figure S10.** ((A, B), (F, G), and (K, L)) IFFT, (inset) FFT patterns, and ((C, D, and E), (H, I, and J) and (M, N, and O)) d spacing line profiles of Se-CoOTe, S-CoOTe, and P-CoOTe respectively.

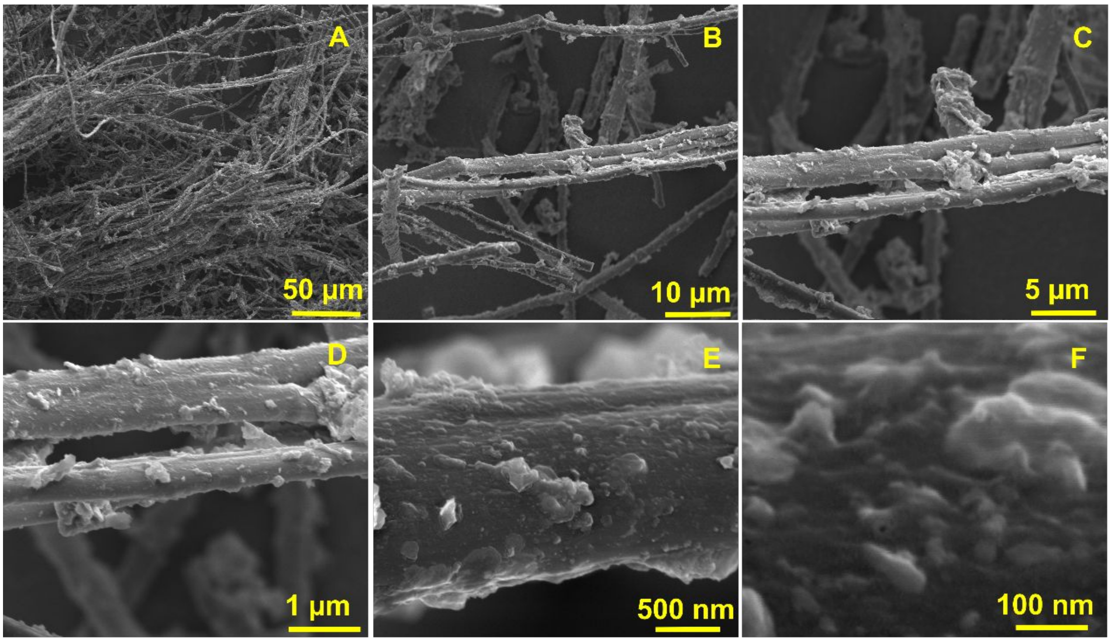


**Figure S11.** (A-F) Different magnified FESEM images of pre-carbonized_C.

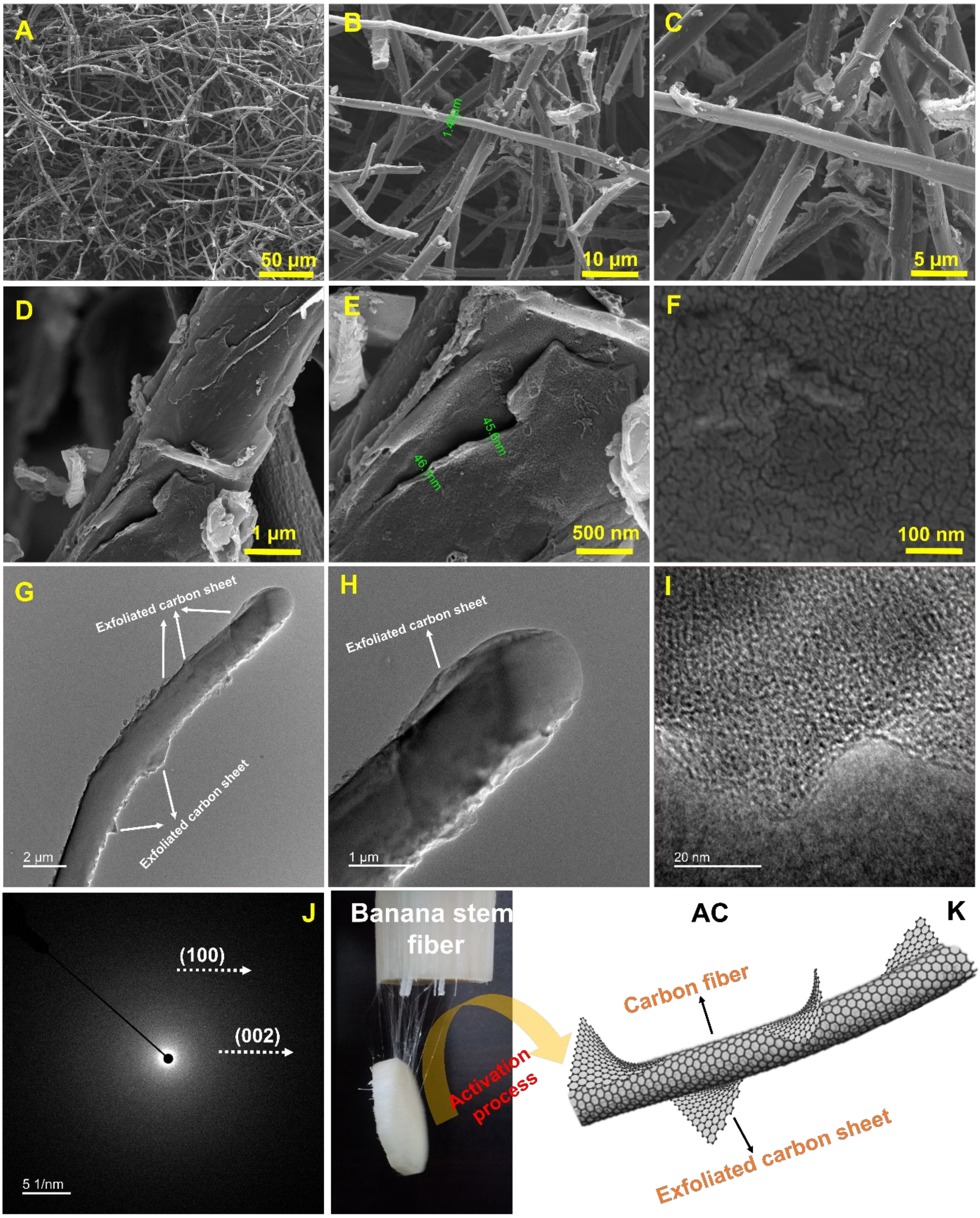


**Figure S12**. (A-F) Different magnified FESEM images, (G, H) TEM images, (I) HR-TEM image, (J) SAED pattern, and (K) photographic and schematic structure of AC.

Figure S11 (A-F) demonstrates the different magnified FESEM images of pre-carbonized_C, which exhibits a fiber-like structure with a longer length and ununiform surface. Meanwhile, the AC (Figure S12 (A-F)) displays a similar fiber-like structure with shorter length, exfoliated graphene sheets with thickness from ~ 45.6 to 46.1 nm, and cracked texture, which is more

favorable for high surface area, good solubility, and better hydrophilicity properties. The TEM images (Figure S12 (G, H)) also mimic the similar results of the above FESEM analysis. As seen in HRTEM (Figure S12I), the AC is the amorphous hard carbon with the presence of a rich amount of super microporosity. The hard carbon and super microporosity natures facilitate more ion diffusion and subsequent interlayer formation during the charge/discharge reaction. It can be further evidenced by observing the two rings in SAED pattern (Figure S12J) for (002) and (100) planes, which are well associated with the amorphous hard carbon. Figure S13 and Figure S14 show the uniform elemental distribution, line mapping, and quantitative result of C and O in both pre-carbonized_C and AC respectively. It suggests that pre-carbonized_C and AC formed without any other impurity. Figure S12K shows the photographic representation of banana core fiber-derived carbon with exfoliated graphene sheets by using a chemical activation process.

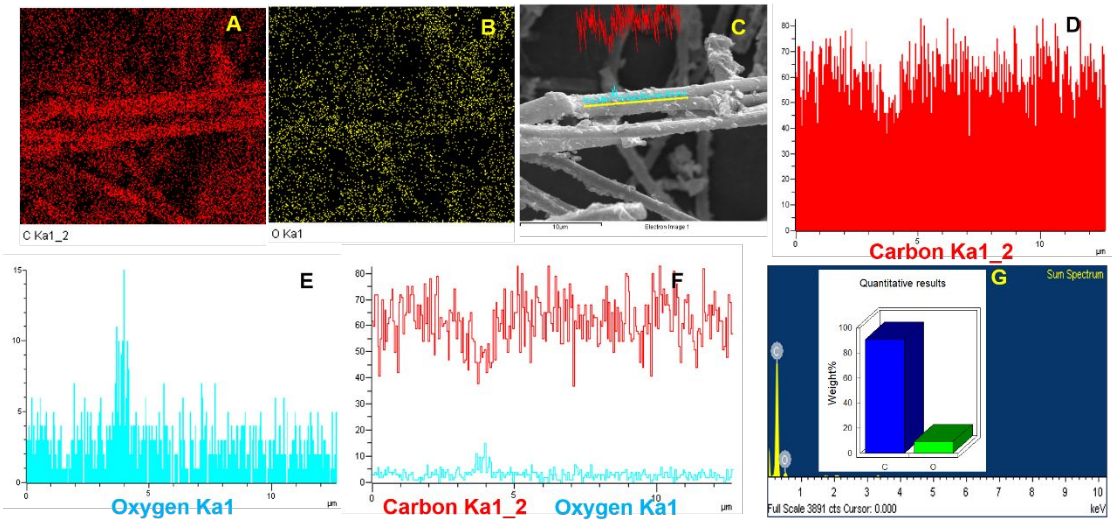


**Figure S13.** (A, B) EDX mapping, (C) FESEM image with line mapping data, (D-F) corresponding line mapping spectra, and (G) EDX spectra with quantitative result of pre-carbonized_C.

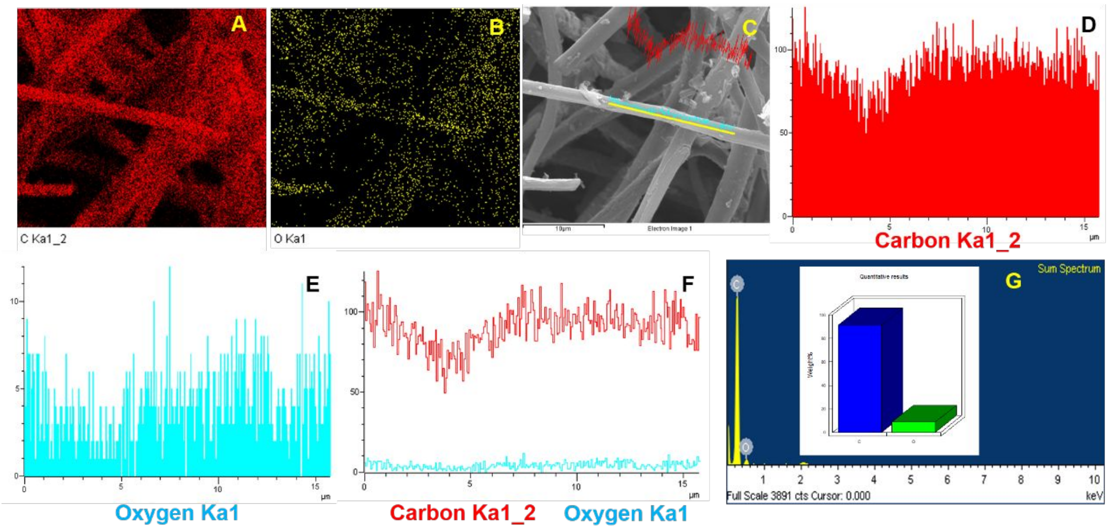


**Figure S14.** (A, B) EDX mapping, (C) FESEM image with line mapping data, (D-F) corresponding line mapping spectra, and (G) EDX spectra with quantitative result of AC.

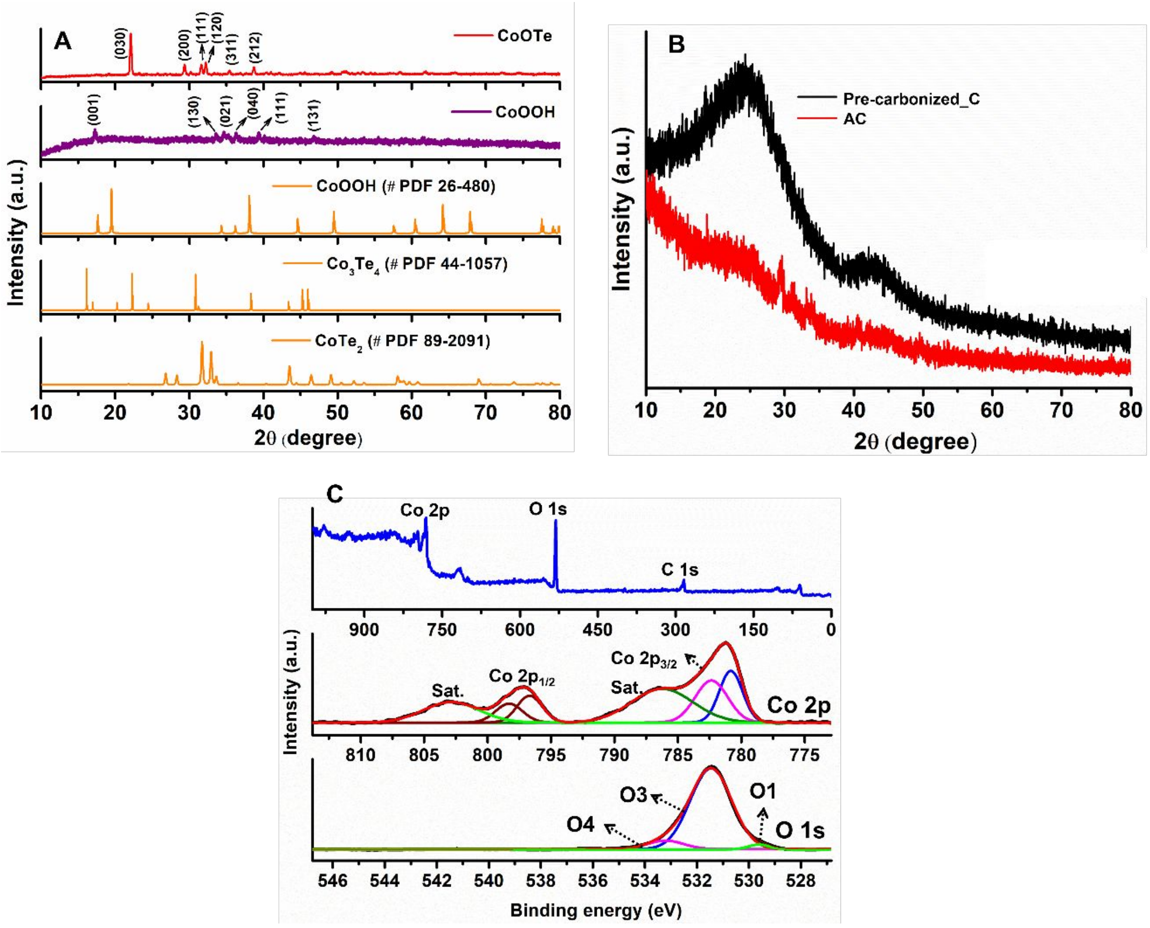


**Figure S15.** (A) XRD patterns of CoOOH and CoOTe and (B) pre-carbonized and AC. (C) XPS spectra of CoOOH.

Figure S15B shows the XRD pattern of pre-carbonized_C and AC with major characteristic peaks of graphitic structure at 24.38° and 42.61° for planes (002) and (100) respectively. The peak of (002) plane exhibits broader and shifts to a lower angle in the XRD pattern of AC, which indicates the formation of disordered amorphous carbon due to the strong KOH chemical activation process.

As seen in high-resolution XPS spectra, the Co 2p element exhibits the two major peaks at 781.22 eV and 797.20 eV for Co $2p_{3/2}$ and Co $2p_{1/2}$ respectively. These two major peaks are further deconvoluted into $Co^{2+}$ $2p_{3/2}$, $Co^{3+}$ $2p_{3/2}$, $Co^{2+}$ $2p_{1/2}$, and $Co^{3+}$ $2p_{3/2}$ at 780.82 eV, 782.42 eV, 796.68 eV, and 798.36 eV respectively. The obtained result indicates the co-existence of $Co^{2+}$ and $Co^{3+}$ oxidation states[3]. The observed satellite peaks at 786.30 eV and 803.07 eV can be associated with the photoionization of the ejected electron from the core of an atom. In general, the core level of O 1s spectrum is divided into four categories such as O1, O2, O3, and O4 for metal-to-oxygen bonding, oxygen vacancy, hydroxyl species, and adsorobed $H_2O$ molecule respectively[4]. The O 1s spectrum of CoOOH shows peaks at 529.58 eV, 531.42 eV and 533.20 eV for O1, O3 and O4 respectively. Herein, the high-intensity peak of O3 represents the hydroxide environment on the surface of CoOOH[5].

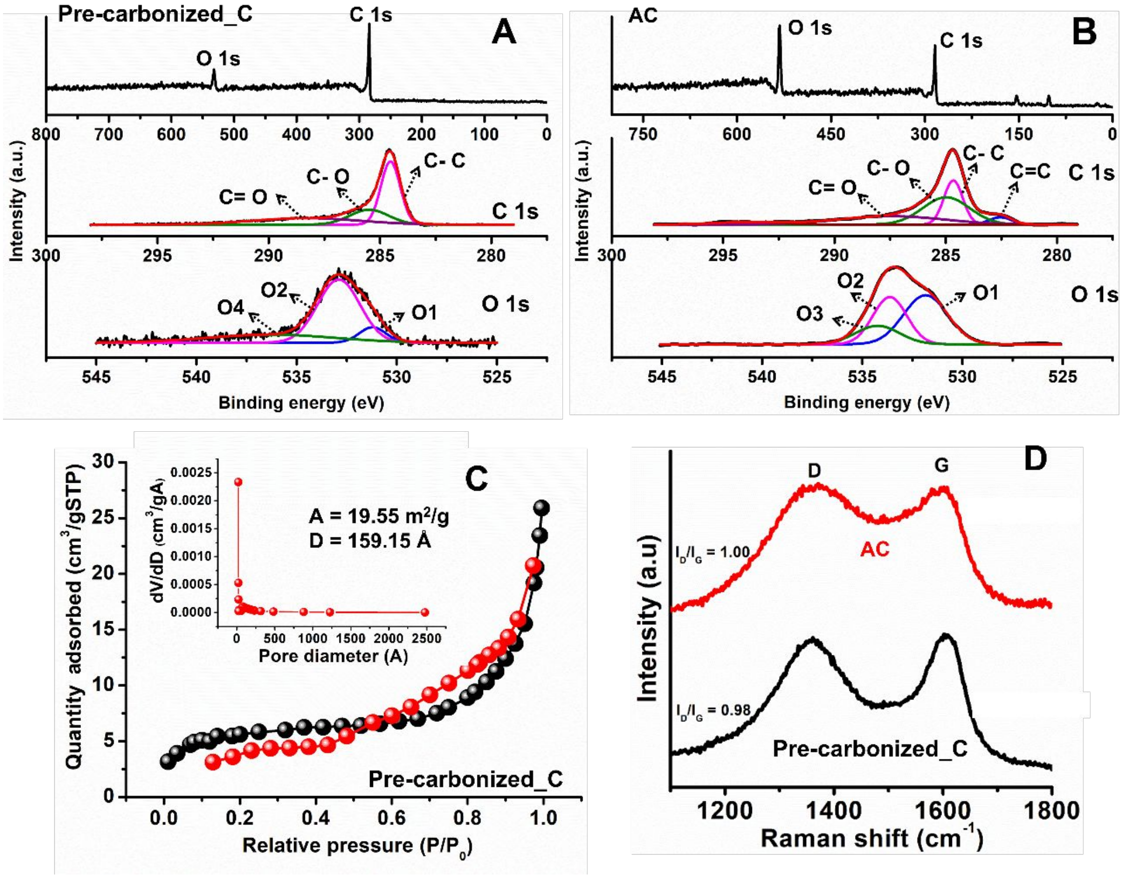


**Figure S16.** (A, B) XPS data, (C) BET isotherm profile and (inset) pore size profile, and (D) Raman spectra of pre-carbonized_C and AC.

The survey spectra of both materials show the major peaks for C 1s and O 1s. However, the intensity of the O 1s peak is higher for AC than that of pre-carbonized_C. The imparted oxygen functional groups facilitate the additional active sites for ion interaction during charge/discharge processes. The high-resolution XPS spectra of C 1s in pre-carbonized_C show the peak for C-C, C-O, and C=O at 284.53 eV, 285.40 eV, and 288.36 eV respectively. In the case of AC, it can be observed that the additional peak for C=C at 282.53 eV and C-O look broader due to the stronger carbonization and activation processes[6]. In addition, both pre-carbonized_C and AC show the peak response of O1 (C=O), O2 (O-C=O/-OH), O3 (O-C=O/C-O), and O4 (adsorbed oxygen) in O 1s spectra[7]. Herein, AC shows the strong intensive peak of O1, O2, and O3, it also indicates the imparted oxygen moieties into the lattice of carbon. Meanwhile, pre-carbonized_C shows O1, O2, and O4 signal responses.

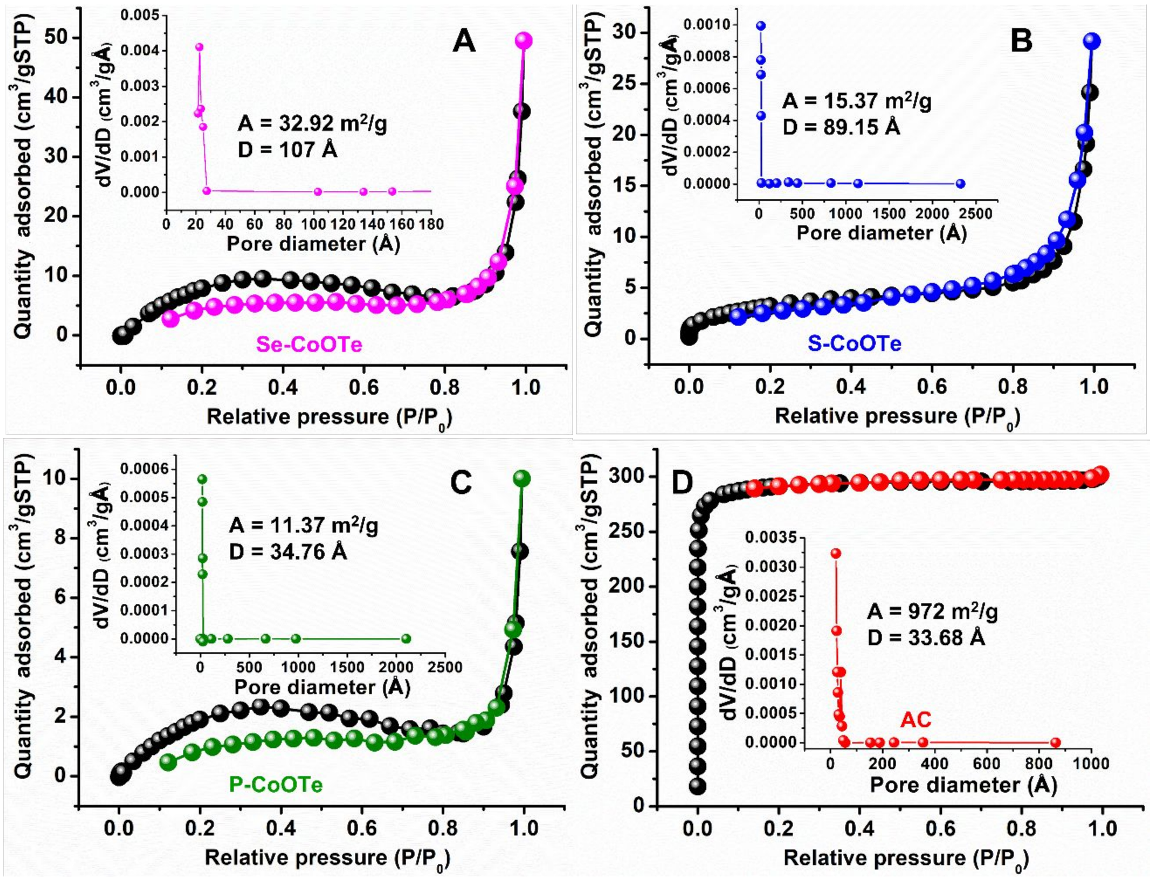


**Figure S17**. BET isotherm (A) Se-CoOTe, (B) S-CoOTe, (C) P-CoOTe, and (D) AC. (inset) Corresponding pore size distribution profile.

As seen in Figure S17 (A-C), the Se-CoOTe exhibits a higher specific surface area (A) of 32.92 $m^2/g$ and pore diameter (D) of 107 Å. It is comparatively higher than those of S-CoOTe (A = 15.37 $m^2/g$ and D = 89.15 Å) and P-CoOTe (A = 11.37 $m^2/g$ and D = 34.76 Å). Herein, Se-CoOTe, S-CoOTe, and P-CoOTe exhibit the Type-IV isotherm and mesoporous characteristics. On the other

hand, Figure S17D shows the BET isotherm of AC with higher A = 972 $m^2/g$ and D = 33.68 Å, which is 48.71 fold higher surface area than those of pre-carbonized_C (A = 19.55 $m^2/g$ and D = 159.15 Å) (Figure S16C). Based on BHJ pore distribution analysis, both pre-carbonized_C and AC exhibit the mesoporous characteristic.

In general, the G band represents the $sp^2$-bonded carbon atom in a graphitic structure, while the D band denotes the defect/disorder in a hexagonal graphitic structure. Herein the $I_D/I_G$ ratio is calculated to evaluate the degree of graphitization and defect formation. According to this hypothesis, the Raman spectra were recorded for pre-carbonized_C and AC and demonstrated in Figure S16D. In this spectra, the intensity of the D band is slightly higher for AC with the $I_D/I_G$ ratio of 1, meanwhile the pre-carbonized_C exhibits the $I_D/I_G$ ratio of 0.98. It again confirmed that the KOH activation creates the defective structure in AC.

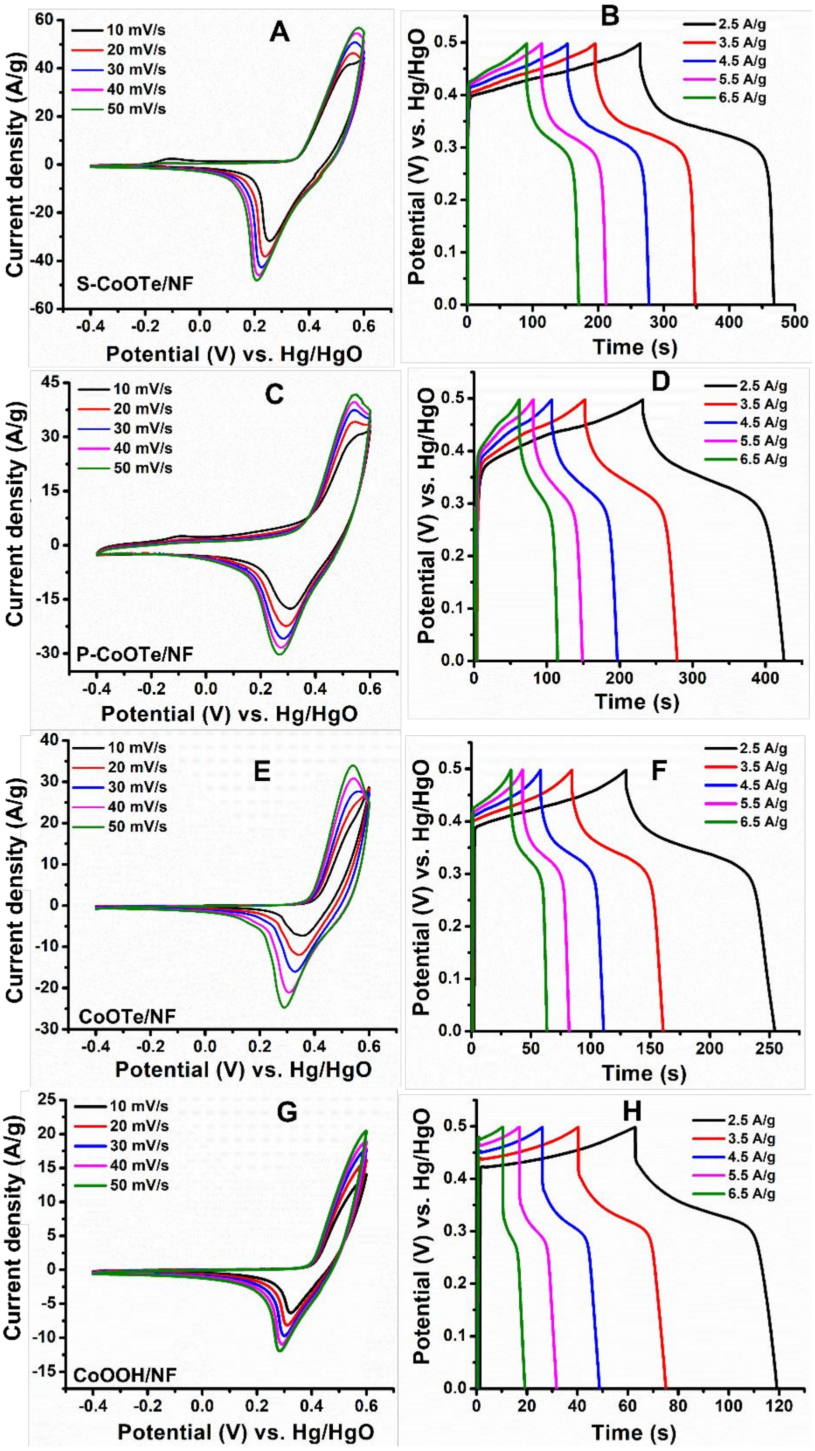


**Figure S18.** CV curves for different scan rate and GCD curves for different current density at (A, B) S-CoOTe/NF, (C, D) P-CoOTe/NF, (E, F) CoOTe/NF, and (G, H) CoOOH/NF.

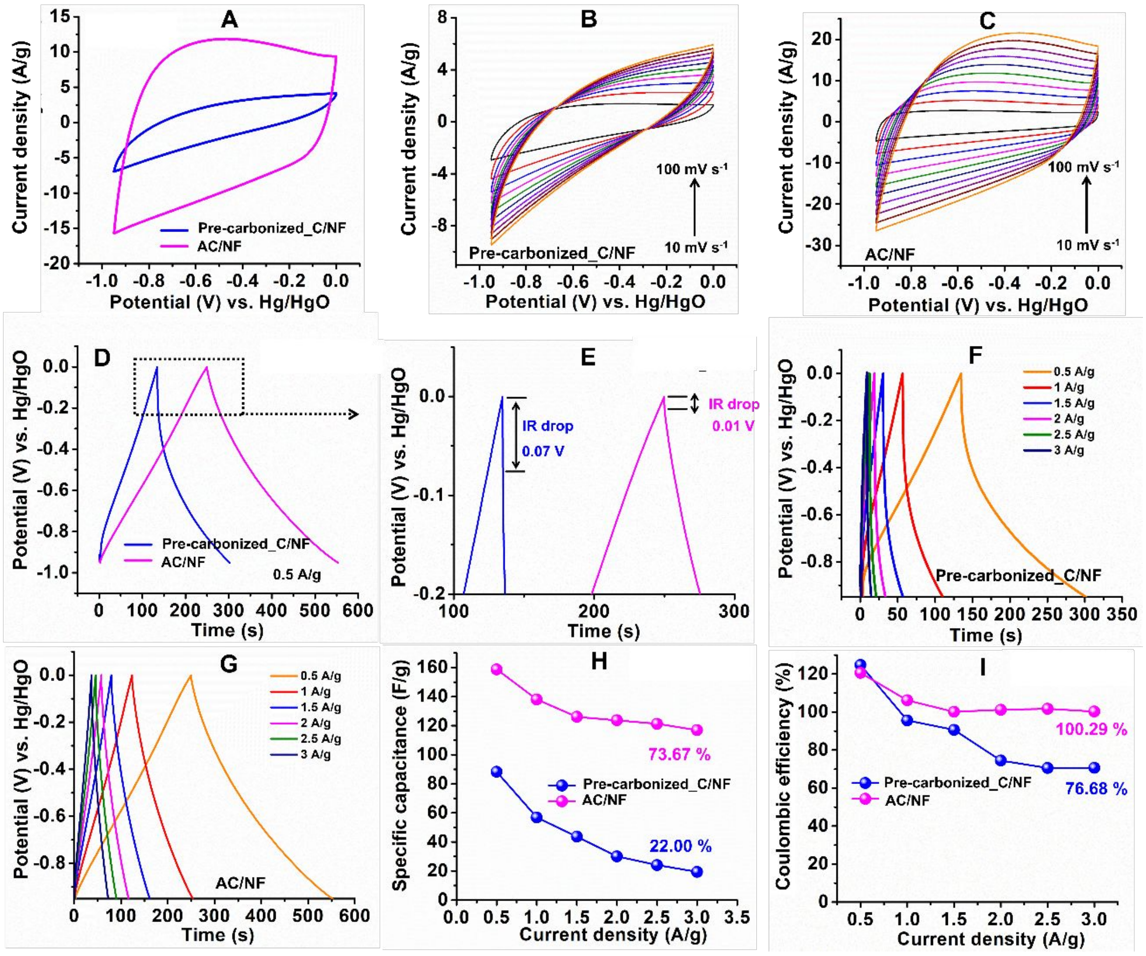


**Figure S19**. CV curves for (A) comparison of different modified electrodes and varying scan rate at (B) pre-carbonized_C/NF and (C) AC/NF. GCD for (D, E) comparison of different modified electrodes and varying current densities at (F) pre-carbonized_C/NF and (G) AC/NF. Corresponding calibration plot for (H) specific capacitance of various electrodes vs. current density, and (I) Coulombic efficiency vs. current density.

Figure S19A shows the rectangular CV curves of pre-carbonized_C/NF and AC/NF with an applied potential window in the range from 0 to -0.96 and a fixed scan rate of 50 mV $s^{-1}$. In general, the rectangular CV curve refers to a non-faradic or electrostatic charge storage reaction. As seen in this result, the AC/NF exhibits a higher integrate CV area of 0.018, while pre-carbonized_C/NF shows about 0.005. The higher integrate CV area indicates the better charge storage performance of AC/NF compared to pre-carbonized_C/NF. And also, the CV curves for different scan rates (10 to 100 mV $s^{-1}$) at pre-carbonized_C/NF and AC/NF are demonstrated in Figure S19(B, C). In these CV results, the integrated area increased for increasing scan rate, which implies the ideal electric double layer characteristic and capacitive behaviors. The GCD curve of pre-carbonized_C/NF and AC/NF (Figure S19D) delivered the symmetric triangular charge/discharge response. It also confirms the double-layer charge storage property of the proposed electrodes. From this plot, it can be observed that AC/NF exhibits the larger charge/discharge time and corresponding higher specific capacitance

(158.7 F/g), which is 0.8 fold higher than pre-carbonized_C (88.7 F/g). Moreover, Figure S19E shows the zoomed image of Figure S19D to estimate the IR drop of the active materials. As seen in this data, the IR drop was calculated to be 0.01 V and 0.07 V for AC/NF and pre-carbonized_C/NF respectively. It suggests higher conductivity and less energy loss at AC/NF electrode during the charge/discharge process. The GCD curves of AC/NF and pre-carbonized_C/NF for different current densities (0.5, 1, 1.5, 2, 2.5, and 3 A/g) are given in Figure S19(F, G). Both AC/NF and pre-carbonized_C/NF show decreased charge/discharge time by increasing the applied current density. By using these data, the calibration plot for current density vs. specific capacitance (Figure S19H) and current density vs. coulombic efficiency (CE) (Figure S19I) were plotted.

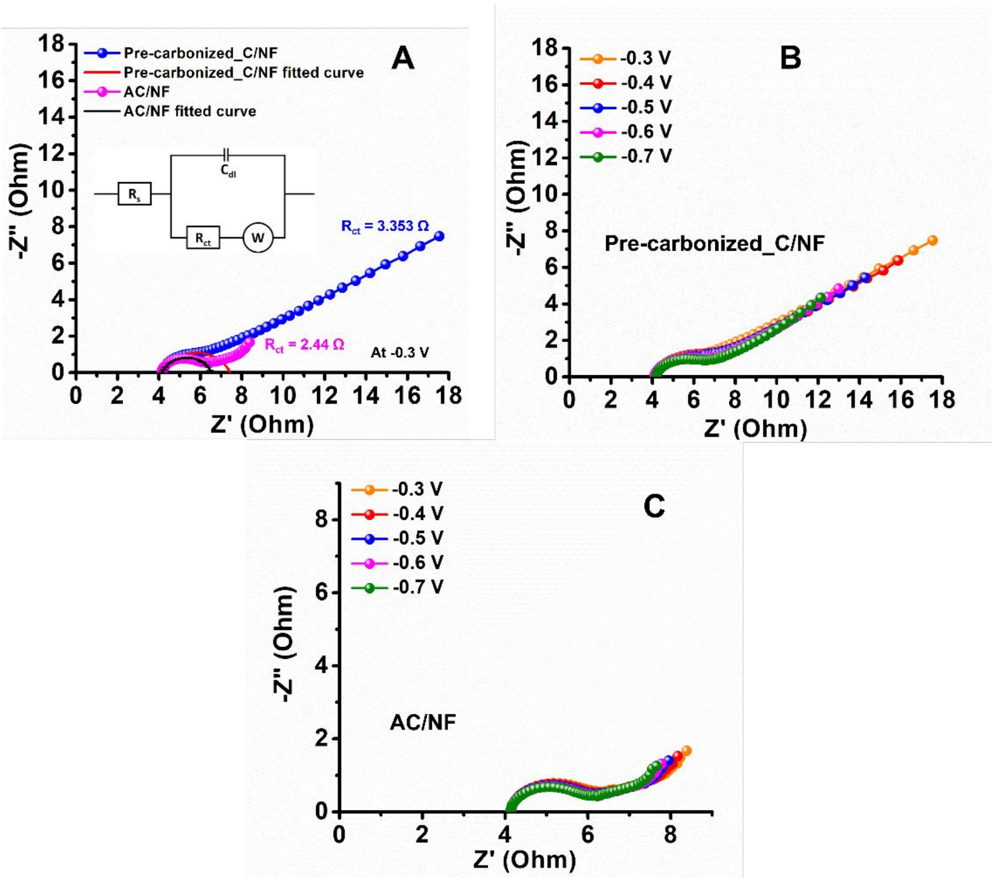


**Figure S20.** (A) Comparison for EIS curve of pre-carbonized_C/NF and AC/NF, (inset) Randles's circuit. EIS curves for different applied potential at (B) pre-carbonized_C/NF and (C) AC/NF.

In the comparison plot (Figure S20A), the fitted Nyquist plot shows the $R_{ct}$ of 2.44 Ω and 3.35 Ω for AC/NF and pre-carbonized_C/NF respectively. In addition, Figure S20(B, C) shows the Nyquist plot for different applied potentials (-0.3, -0.4, -0.5, -0.6, and -0.7) at AC/NF and pre-carbonized_C/NF. Both AC/NF and pre-carbonized_C/NF delivered a very slightly decreased value of $R_{ct}$ for increasing applied potential. It can be related to the polarization of ions on the electrode surface by increasing the applied potential.

For understanding the real-time application of prepared AC fiber, which was compared with commercially available CNF. From Figure S21, it can be observed that both pre-carbonized_C/NF and AC/NF electrodes exhibited the CV curves with larger integrated areas than compared to commercial CNF/NF. It indicates the advantage and suitability of banana stem core fiber derived AC fiber for commercial applications.

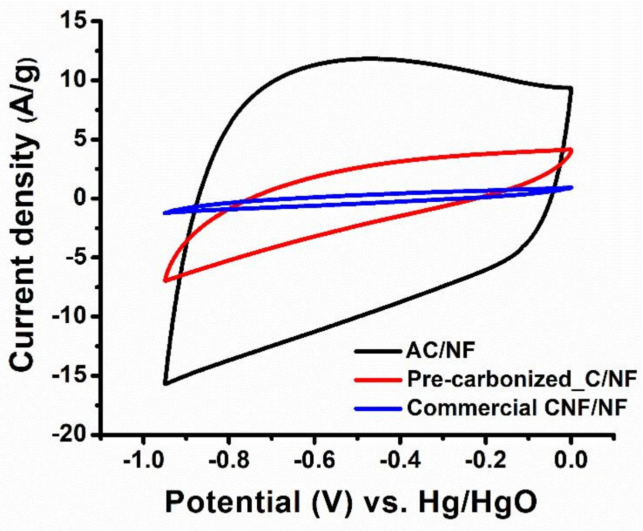


**Figure S21**. Comparison CV curves of AC/NF, pre-carbonized_C/NF, and commercial CNF/NF electrodes at a scan rate of 50 mV $s^{-1}$.

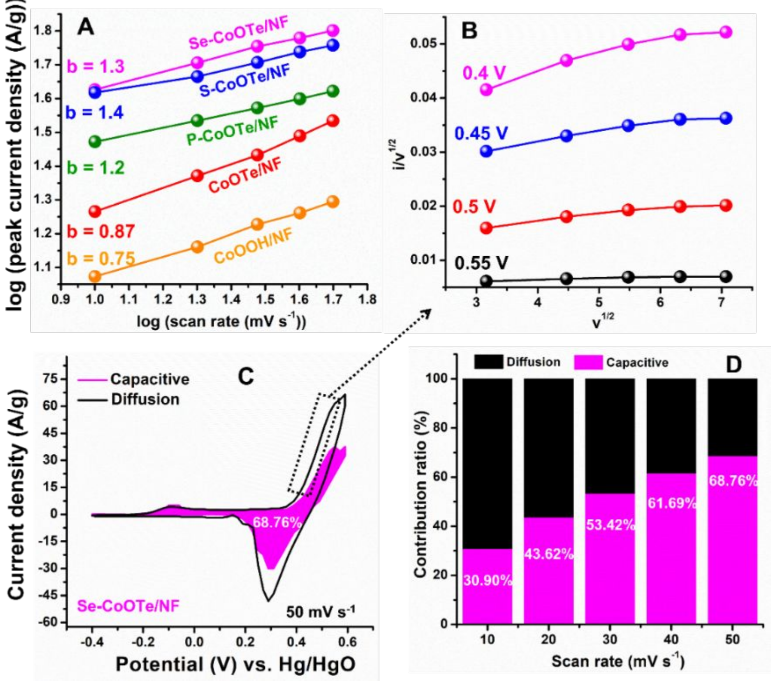


**Figure S22**. (A) Linear calibration plot (log (scan rate (mV $s^{-1}$) vs. log (peak current density (mA/ $cm^2$)) of different modified electrodes. (B) Linear calibration plot for $\nu^{1/2}$ vs. $i/\nu^{1/2}$ at different fixed potential from CV curve of Se-CoOTe/NF. (C) CV curves of Se-CoOTe/NF for capacitive and diffusion ratio. (D) Bar diagram for scan rate vs. contribution ratio (%) of Se-CoOTe/NF.

In the power law approach, the relationship of scan rate (ν) and peak current (i) can be written as the following equation (Eq. S1)

$$i = a\nu^{b} \qquad \text{(Eq. S1)}$$

where, both a and b are appropriate constant values. The b value can be calculated by taking the logarithm on both sides of the above equation and thus obtaining the linear calibration plot for the logarithm of scan rate vs. logarithm of oxidation peak current as shown in Figure S22A. The b values of 0.5 and 1 are the ideal range for diffusion and surface-controlled process respectively.

According to the theory of Dunn and coworkers, the diffusion and capacitive contributions can be quantitatively separated from total charge storage by using the below equations (Eq. S2) and (Eq. S3)

$$i\ (\nu) = k_1\nu + k_2\nu^{1/2} \qquad \text{(Eq. S2)}$$

Equation (S2) can be altered as below

$$i/\nu^{1/2} = k_1\nu^{1/2} + k_2 \qquad \text{(Eq. S3)}$$

where, i represents the current (A) at a certain potential, and ν refers to the scan rate (mV $s^{-1}$). The $k_1\nu$ and $k_2\nu^{1/2}$ represents capacitive and diffusion contributions respectively. The $k_1$ and $k_2$ are both adjustable parameters which can be found from the slope and y-axis intercept point of the linear plot ($\nu^{1/2}$ *vs.* $i/\nu^{1/2}$). Herein, the linear calibration plot ($\nu^{1/2}$ *vs.* $i/\nu^{1/2}$ ) should be plotted at different potentials and sweep rates varied between 10 and 50 mV/s. By following the above procedure, the fraction of the current from capacitive effects and the diffusion-controlled faradaic process can be quantitatively estimated. After performing this calculation under different scan rates, plot the $k_1\nu$ (shaded area) as well as the experimental currents (solid line area) as shown in Figure S22.

In general, the total charge storage of the electrode is described from the total contribution of diffusion and capacitive mechanism. There are two kinds of methods to evaluate the diffusion and capacitive percentages such as the power law relationship and Dunn and coworkers reported approach[8, 9].

Based on power law equation, the b value can be calculated by taking the logarithm on both sides of the above equation and thus obtaining the linear calibration plot for the logarithm of scan rate vs. logarithm of oxidation peak current as shown in Figure S22A. The b value of 0.5 indicates that the total charge storage process follows a semi-infinite diffusion process, while the b value of 1 indicates the complete surface-controlled mechanism. As seen in the calibration plot, CoOOH/NF and CoOTe/NF electrodes exhibit the b value of 0.75 and 0.87 respectively, which are suggesting both diffusion and surface-controlled mechanisms involved in the charge storage process[10].

By following the theory of Dunn and coworkers, $k_1$ and $k_2$ are constants and derived from the linear calibration plot of $i/v^{1/2}$ *vs.* $v^{1/2}$ (Figure S22B). By following this method, the CV profile of capacitive (shade with color) and diffusion (without shade) currents for Se-CoOTe/NF at a scan rate of 50 mV $s^{-1}$ were plotted as shown in Figure S22C.

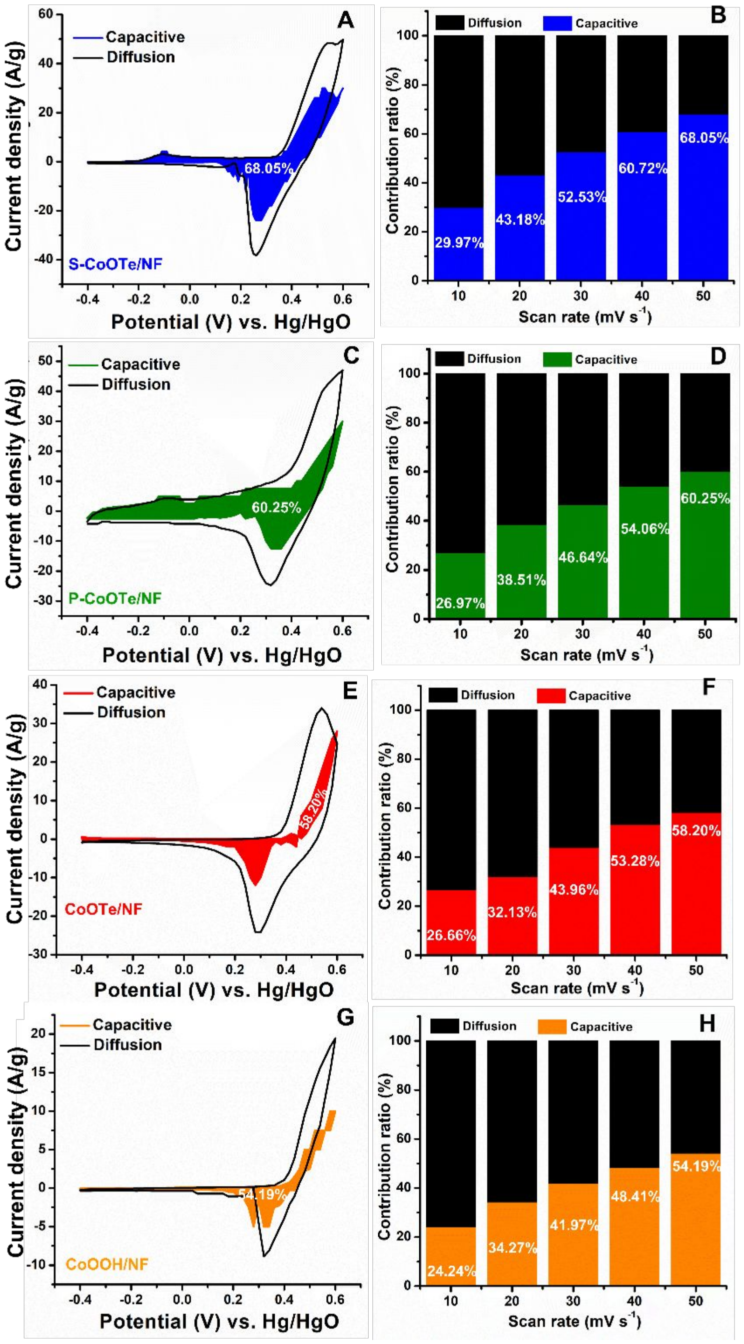


**Figure S23.** CV curves and bar diagram for capacitive and diffusion ratio of (A, B) S-CoOTe/NF, (C, D) P-CoOTe/NF, (E, F) CoOTe/NF, and (G, H) CoOOH/NF.

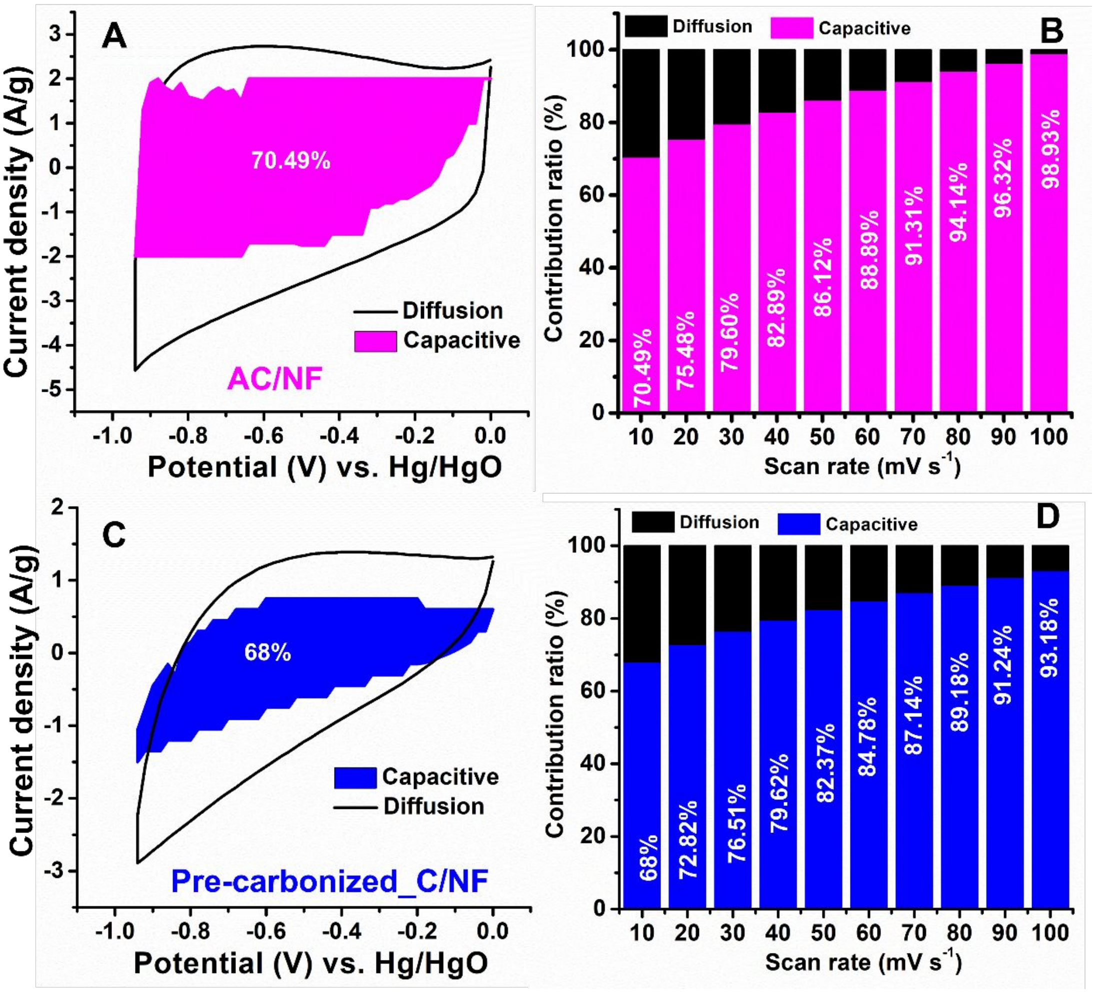


**Figure S24**. (A) CV curves of AC/NF modified electrode for capacitive and diffusion ratio and (B) corresponding bar diagram for scan rate vs. contribution ratio. (C) CV curves of pre-carbonized_C/NF modified electrode for capacitive and diffusion ratio and (D) corresponding bar diagram for scan rate vs. contribution ratio.

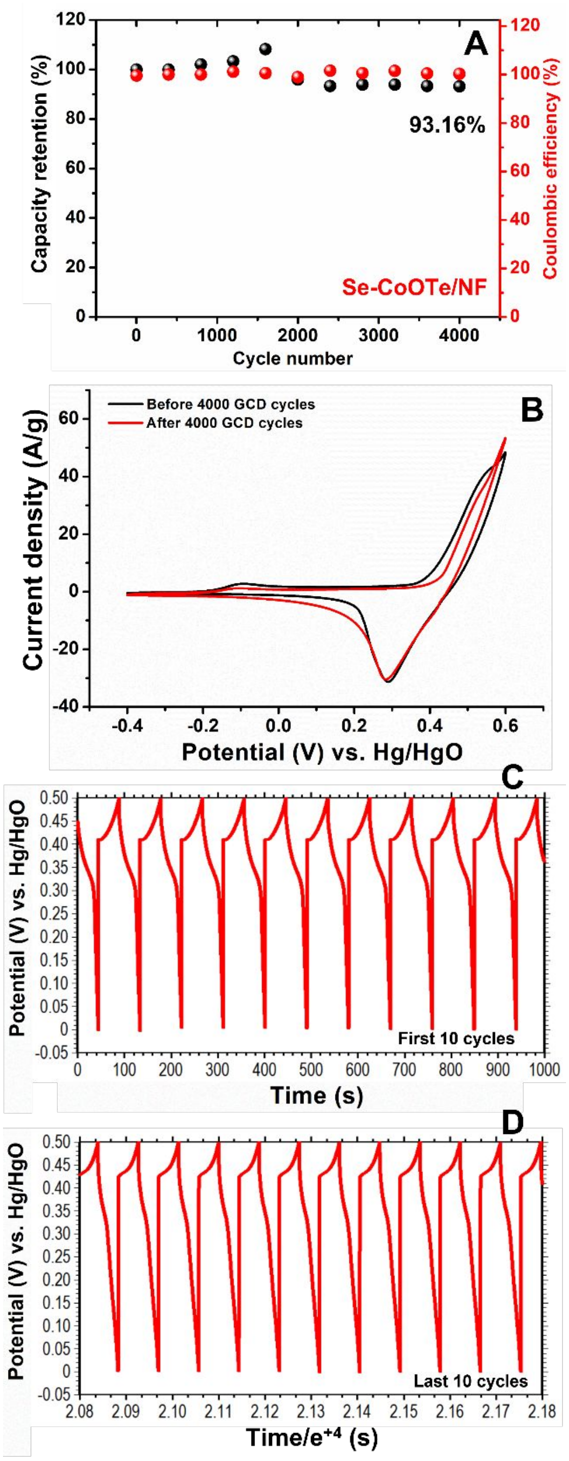


**Figure S25.** (A) Calibration plot for long term stability and Coulombic efficiency of Se-CoOTe/NF. (B) Comparison of CV curves for before and after long term stability test. (C, D) GCD curves of first 10 cycles and last 10 cycles from 4,000 cycles.

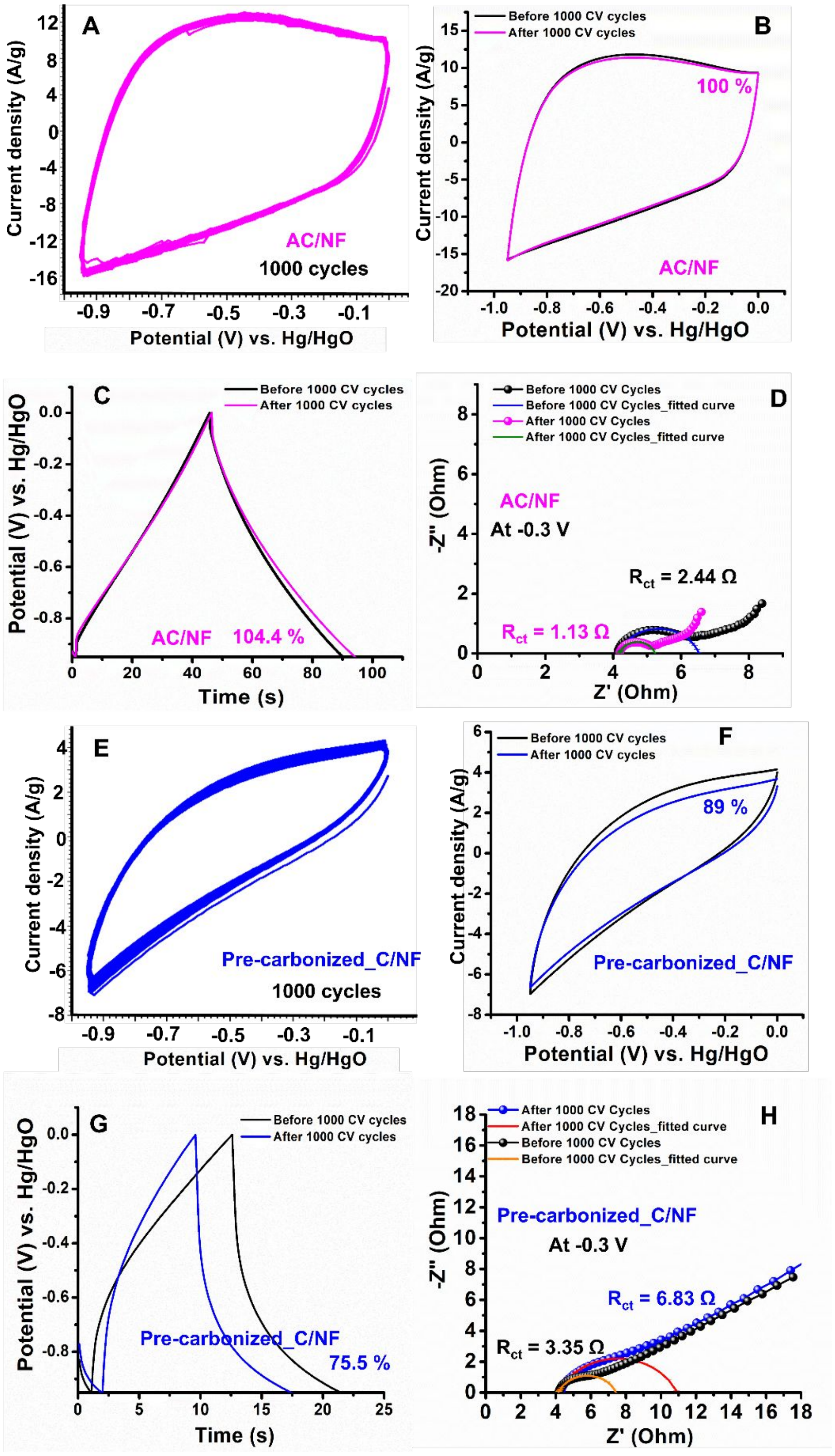


**Figure S26.** (A) CV curve for continuous 1,000 cycles, comparison of (B) CV, (C) GCD, (D) EIS curves for before and after 1,000 cycles of AC/NF. (E) CV curve for continuous 1000 cycles, comparison of (F) CV, (G) GCD, (H) EIS curves for before and after 1,000 cycles of pre-carbonized C/NF.

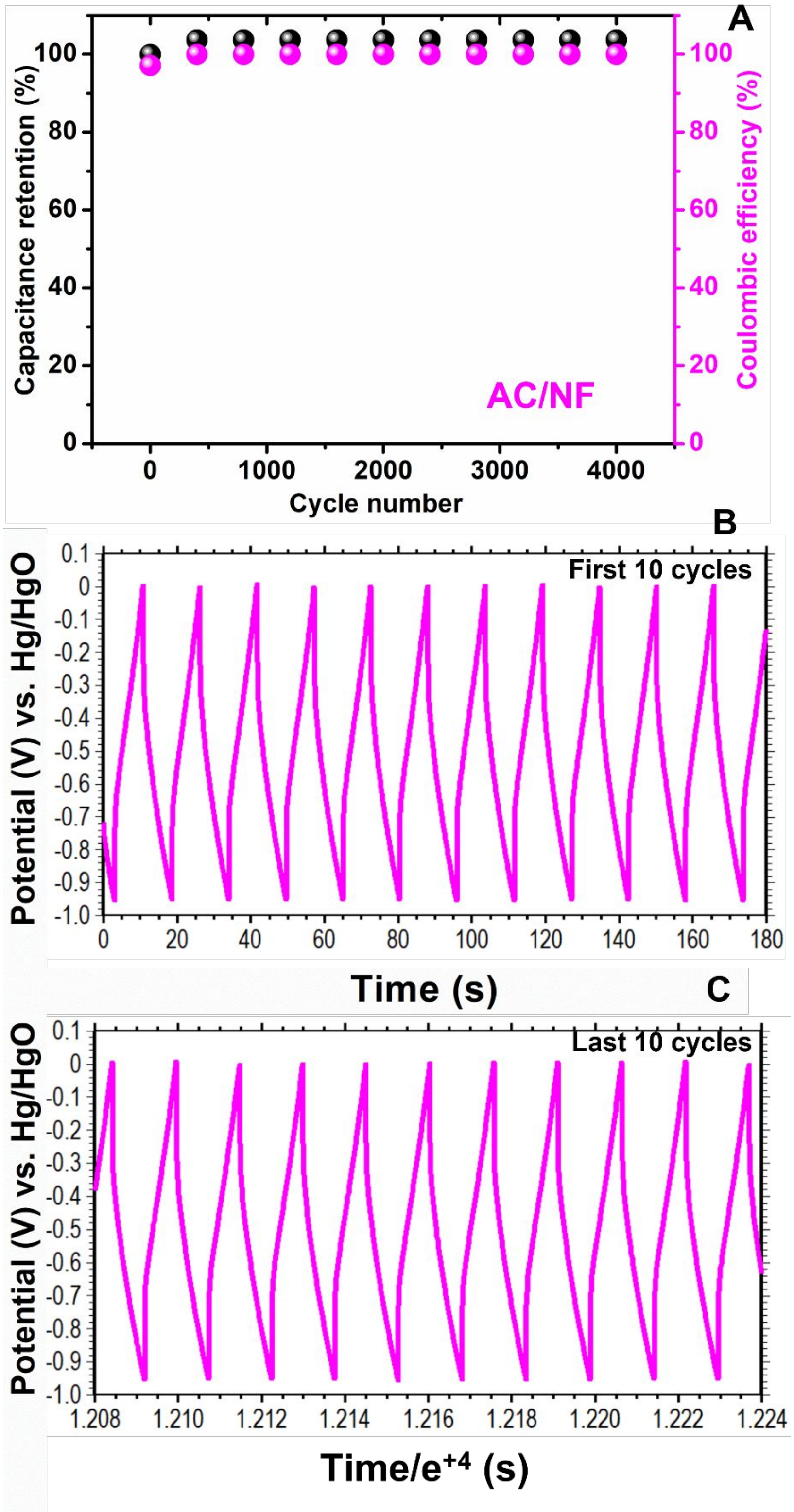


**Figure S27.** (A) Calibration plot for long term stability and Coulombic efficiency of AC/NF. (B, C) GCD curves of first 10 cycles and last 10 cycles from 4,000 cycles.

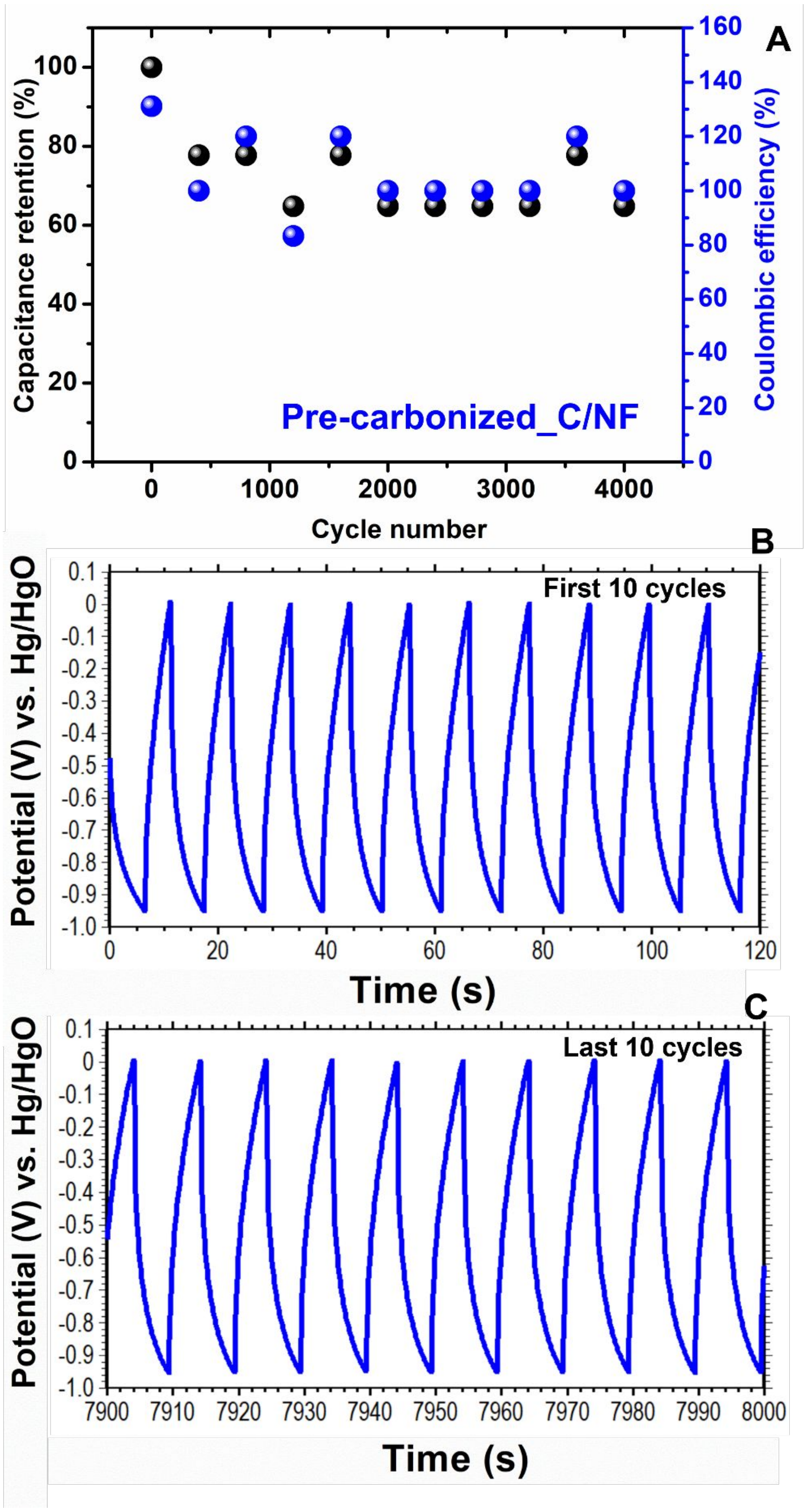


**Figure S28.** A) Calibration plot for long term stability and Coulombic efficiency of pre-carbonized C/NF. (B, C) GCD curves of first 10 cycles and last 10 cycles from 4,000 cycles.

The CV shape of AC/NF remains similar without wide deviation compared to pre-carbonized_C/NF. The corresponding CV curves for before and after 1000 cycles confirm the 100% higher stability of AC/NF (Figure S26B), while pre-carbonized_C/NF (Figure S26F) delivered 89%. The comparison of GCD curves before and after 1000 cycles are revealed in Figure S26 (C, G). From this result, the AC/NF delivered an almost analogous GCD curve and retains 104.4% discharge time, while pre-carbonized_C/NF delivered only 75.5%. For additional validation, the EIS study also executed and presented the corresponding Nyquist plot before and after 1000 cycles. The $R_{ct}$ value of AC/NF (Figure S26D) decreased from 2.44 to 1.13 Ω after the cycle test. As given in Figure S26H, the $R_{ct}$ value of pre-carbonized_C/NF alternatively increases from 3.35 to 6.83 Ω. It strongly recommends the superior stability and reversibility of AC/NF compared to pre-carbonized_C/NF. The GCD cycle stability experiment was performed for AC/NF and pre-carbonized_C/NF electrodes by applying the fixed current density of 3 A/g and 4000 continuous charge/discharge cycles. The AC/NF delivered almost 100% of capacitance retention and coulombic efficiency (Figure S27A) owing to higher stability. At the same time, the data point for capacitance retention and coulombic efficiency of pre-carbonized_C/NF (Figure S28A) show more fluctuation. In fact, the GCD curves for the first and last 10 cycles of both AC/NF (Figure S27 (B, C)) and pre-carbonized_C/NF (Figure S28 (B, C)) retain a similar shape due to the better reversibility. The obtained GCD cycle stability results also mimic the CV cycle stability test results.

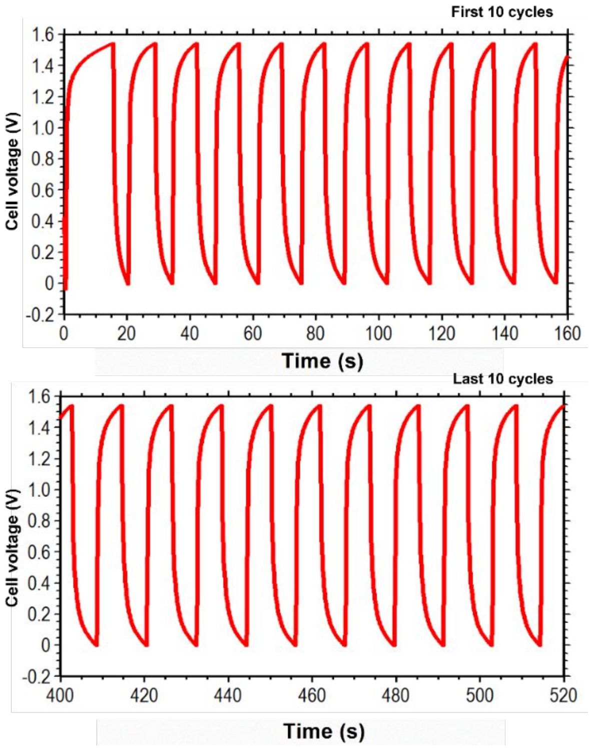


**Figure S29**. GCD curves of first 10 cycles and last 10 cycles from 10000 cycles of asymmetric device.

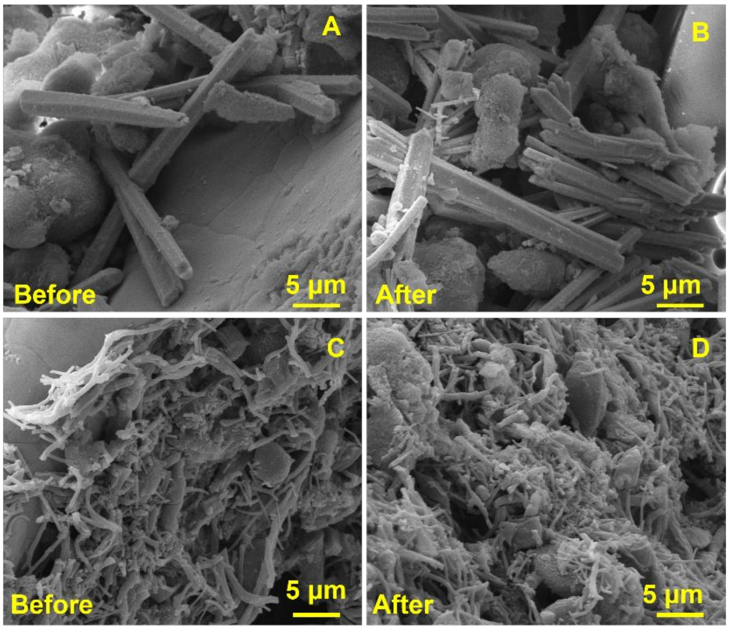


**Figure S30**. SEM images of (A, B) Se-CoOTe/NF and (C, D) AC/NF for before and after long-term stability test.

## References

(1) Yang, B.; Zuo, X.; Yang, X.; Zhou, L.; Li, G. Effects of pH Values on Crystal Growth and Photoluminescence Properties of ZnO Hexagonal Rods with Cones. *Mater. Lett.* **2014**, 130, 123-126.

(2) Wahab, R.; Hwang, I. H.; Kim, Y. S.; Shin, H. S. Photocatalytic Activity of Zinc Oxide Micro-flowers Synthesized via Solution Method. *Chem. Eng. J.* **2011**, 168, 359-366.

(3) Kalasina, S.; Kongsawatvoragul, K.; Phattharaphuti, P.; Phattharasupakun, N.; Sawangphruk, M. Cobalt Oxysulphide/hydroxide Nanosheets with Dual Properties Based on Electrochromism and a Charge Storage Mechanism. *RSC Adv.* **2020**, 10, 14154-14160.

(4) Wang, C. C.; Liu, M.; Man, B. Y.; Chen, C. S.; Jiang, S. Z.; Yang, S. Y.; Gao, X. G.; Xu, S. C.; Hu, B.; Sun, Z. C.; Guo, J. J.; Hou, J. Role of Cobalt in Room-Temperature Ferromagnetic Co-doped ZnO Thin Films, *AIP Adv.* **2012**, 2, 012182-012189.

(5) Burke, M. S.; Kast, M. G.; Trotochaud, L.; Smith, A. M.; Boettcher, S. W. Cobalt−Iron (Oxy)Hydroxide Oxygen Evolution Electrocatalysts: The Role of Structure and Composition on Activity, Stability, and Mechanism. *J. Am. Chem. Soc.* **2015**, 137, 3638-3648.

(6) Xu, Q.; Chen, J.; Pu, P.; Liu, Y.; Zhao, J.; Dong, C.; Gao, C.; Chen, Y.; Zhou, H. Preparation of Highly Photoluminescent Sulfurdoped Carbon Dots for Fe(III) Detection. *J. Mater. Chem. A* **2015**, 3, 542-546.

(7) Chen, W.; Gong, M.; Li, K.; Xia, M.; Chen, Z.; Xiao, H.; Fang, Y.; Chen, Y.; Yang, H.; Chen, H. Insight into KOH Activation Mechanism During Biomass Pyrolysis: Chemical Reactions Between O-Containing Groups and KOH, *Appl. Energy* **2020**, 278, 115730-115748.

(8) Wang, R.; Wang, S.; Peng, X.; Zhang, Y.; Jin, D.; Chu, P. K.; Zhang, L. Elucidating the Intercalation Pseudocapacitance Mechanism of MoS−Carbon Monolayer Interoverlapped Superstructure: Toward High-Performance Sodium-Ion-Based Hybrid Supercapacitor. *ACS Appl. Mater. Interfaces* **2017**, 9, 32745-32755.

(9) Liang, M.; Zhao, M.; Wang, H.; Shen, J.; Song, X. Enhanced Cycling Stability of Hierarchical $NiCo_2S_4$@$Ni(OH)_2$@PPy Core–Shell Nanotube Arrays for Aqueous Asymmetric Supercapacitors. *J. Mater. Chem. A* **2018**, 6, 2482-2493.

(10) Guo, F.; Gupta, N.; Teng, X. Enhancing Pseudocapacitive Process for Energy Storage Devices: Analyzing the Charge Transport Using Electro-Kinetic Study and Numerical Modeling, Supercapacitors Theoretical and Practical Solutions, *IntechOpen*, **2018**, *DOI:10.5772/intechopen.73680.*
